# Discovery of Na_v_1.7 Inhibitors through the Screening of Marine Natural Product Extracts

**DOI:** 10.1021/acs.jnatprod.5c00978

**Published:** 2025-10-22

**Authors:** Adetola H. Adewole, Bhuwan Khatri Chhetri, Ghada M. Abdelwahab, Riya Bhanushali, Anne Marie Sweeney-Jones, Madison Greene, Jaehoon Shim, Carter K. Asef, Patric Vaelli, Lee Barrett, Facundo M. Fernández, Cassandra L. Quave, Julia Kubanek

**Affiliations:** † School of Biological Sciences, Georgia Institute of Technology, Atlanta, Georgia 30332, United States; ‡ School of Chemistry and Biochemistry, Georgia Institute of Technology, Atlanta, Georgia 30332, United States; § F.M. Kirby Neurobiology Center, Boston Children’s Hospital, and Department of Neurobiology, 1811 Harvard Medical School, Boston, Massachusetts 02115, United States; ∥ Department of Neurobiology, Harvard Medical School, Boston, Massachusetts 02115, United States; # Parker H. Petit Institute for Bioengineering and Bioscience, Georgia Institute of Technology, Atlanta, Georgia 30332, United States; ⊥ Department of Dermatology, Center for the Study of Human Health, 1371 Emory University, Atlanta, Georgia 30322, United States

## Abstract

Automated high-throughput screening of a prefractionated extract library of marine macroorganisms identified 239 hits (hit rate 2.5%), including a marine algal extract that blocked the Na_v_1.7 channel in a fluorescent-based flux assay. Bioactivity-guided chemical investigation led to the isolation of two glycoglycerolipids (**1** and **2**). In preliminary screening, **1** showed stronger Na_v_1.7 inhibition, while secondary screening using patch-clamp electrophysiology, which measures ion movement across membranes, revealed **2** as more potent. This study identified some ion channel modulators from diverse taxonomic origins, including red algae, sponges, and corals, many of which are underexplored and represent promising leads for future drug discovery.

## Graphical abstract

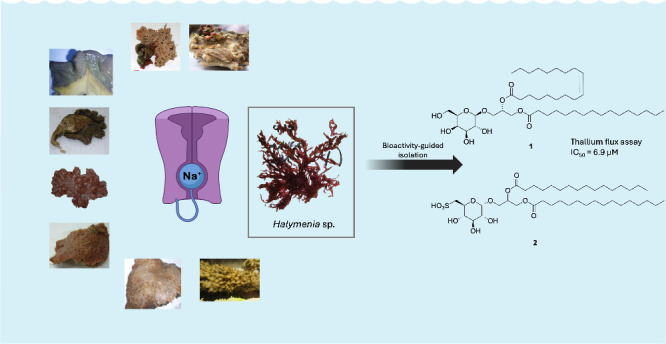

Voltage-gated ion channels are transmembrane proteins that play a key role in generating and propagating electrical signals. Their activity is controlled by changes in membrane potentials, with channel opening allowing the movement of ions.^1–3^ Voltage-gated sodium channels (Na_v_), a class of transmembrane ion channels, mostly found in the peripheral nervous system, are promising targets for analgesic activity.^4^ Their exploitation presents a potential alternative to opioids, which act via interaction with G-protein coupled receptors and are subject to addiction and overdose risks.^5^

Natural products have played a pivotal role in drug discovery,^6^ and previous studies have screened natural product extracts for small molecules targeting voltage-gated ion channels. Na_v_1.7 and Na_v_1.8 are voltage-gated sodium channel isoforms with distinct contributions to pain signaling. Na_v_1.7 acts as a threshold channel that initiates action potential, whereas Na_v_1.8 mediates repetitive firing and thereby sustains their propagation.^7–9^ Cai et al.^10^ reported that two clerodane diterpenoids, (−)-hardwickiic acid and hautriwaic acid, isolated from the aerial parts of *Salvia wagneriana*, a flowery shrub, and *Croton setigerus*, an ornamental plant, respectively, blocked sodium channels without affecting calcium or potassium channels. Naringenin, a flavonoid abundant in citrus fruits, selectively inhibited Na_v_1.8, and Khanna and colleagues^11^ proposed its potential use in pain management. Two meroterpenoids, acetoxydehydroaustin A and austin, isolated from the fungus *Verticillium albo-atrum*, activated sodium currents in the central neurons of the cotton bollworm *Helicoverpa armigera*.^12^ The acetylated derivative of an ent-kaurane diterpenoid fungal metabolite, 1-*O*-acetylgeopyxin A, was also found to inhibit Na_v_1.7.^13^

Beyond sodium channel activity, conotoxins, a group of disulfide-rich, neuropeptides isolated from venomous snails of the genus *Conus*, have been shown to selectively block the voltage-gated calcium channel Ca_v_2.2, making them attractive leads in drug development.^14^ A synthetic derivative of ω-conotoxin, Ziconotide, was approved in 2004 by the FDA as a nonopioid analgesic.^15^ Leconotide, another ω-conotoxin, exhibited potency and high selectivity but failed to advance beyond Clinical Phase IIa trials due to its low safety profile at higher doses.^16,17^ Physalin F, a steroidal derivative from *Physalis acutifolia*, a flowering plant in the Solanaceae family native to the Southwestern United States, exhibited antinociceptive activity in neuropathic pain models by selectively blocking voltage-gated calcium channels Ca_v_2.2 and Ca_v_2.3, without affecting sodium or potassium channels.^18^ Capnellene, a tricyclic sesquiterpene isolated from the soft coral *Capnella imbricata*, was found to induce pain-relieving effects in neuropathic mice models.^19^ In that work, Wen and co-workers attributed the analgesic effect to the substantial inhibition of COX-2 protein expression, a protein that can excite the nerves that transmit pain signals. In their review,^20^ Khanna and colleagues compiled approximately 40 plants and fungi-derived natural products that modulate voltage-gated sodium and calcium channels. They highlighted the structural diversity of these compounds, which include alkaloids, terpenes/terpenoids, phenolics, and flavonoids (including their glycosides). The authors noted that while certain natural products act selectively on sodium channels and others on calcium channels, a subset exhibits activity on both channels.

Natural products modulate ion channels through diverse mechanisms. Conotoxins from cone snail venoms, saxitoxins from shellfish, and tetrodotoxin from pufferfish act as antagonists by blocking voltage-gated channels.^21–24^ In contrast, batrachotoxins from poison dart frogs and the diterpenoid alkaloid aconitine function as agonists by keeping sodium channels in an open state.^25–27^ Furthermore, veratridine, a naturally occurring neurotoxin, was proposed to act as an allosteric modulator, altering channel activity without binding to the primary active site.^28^

The marine environment’s rich biodiversity represents an untapped resource for novel chemical scaffolds, with ongoing studies aimed at discovering natural products with potent pharmacological properties. While previous investigations into sodium channel modulators have examined both terrestrial and marine sources, our study focused on marine extracts with the aim of identifying novel sodium channel inhibitors, thereby expanding the chemical space.

In our pursuit of biologically active secondary metabolites, selected natural product mixtures from our International Cooperative Biodiversity Group (ICBG) library of over 9000 unique marine algal and invertebrate extract fractions collected from Fiji and the Solomon Islands were screened against a voltage-gated sodium channel assay as a potential pain target.^29^ This broad screening approach was employed to explore their potential activity as sodium channel inhibitors. Our goal was to identify hits that could establish how prevalent sodium channel modulators are among marine natural products, serving as a starting point for further investigation. The tropical red alga *Halymenia* sp., an understudied species in the Halymeniaceae family, was prioritized among hits for bioactivity-guided fractionation to discover Na_v_1.7 blockers, leading to the isolation of two bioactive glycoglycerolipids. This selection was further motivated by our field observations, which revealed relatively intact tissues despite the genus being recognized as a food source, suggesting the presence of deterrent secondary metabolites, as well as by the limited knowledge of its chemistry.

## Results and Discussion

As part of our ongoing efforts to identify and isolate biologically active and/or novel natural products, 9360 midpolar prefractionated extracts from 3238 marine organisms in the Georgia Tech ICBG library were prioritized based on taxonomic diversity and subjected to high-throughput screening focused on ion channel modulation. In the preliminary screen, extract fractions were evaluated using a fluorescence-based thallium (Tl^+^) flux assay, targeting the sodium channel Na_v_1.7, expressed in a stable cell line. Extract fractions exhibiting significant Na_v_1.7 blocking activity and a z-score of −2 or lower were classified as hits (Figure 1). Using these criteria, a hit rate of 2.5% was observed.

**Figure 1:**
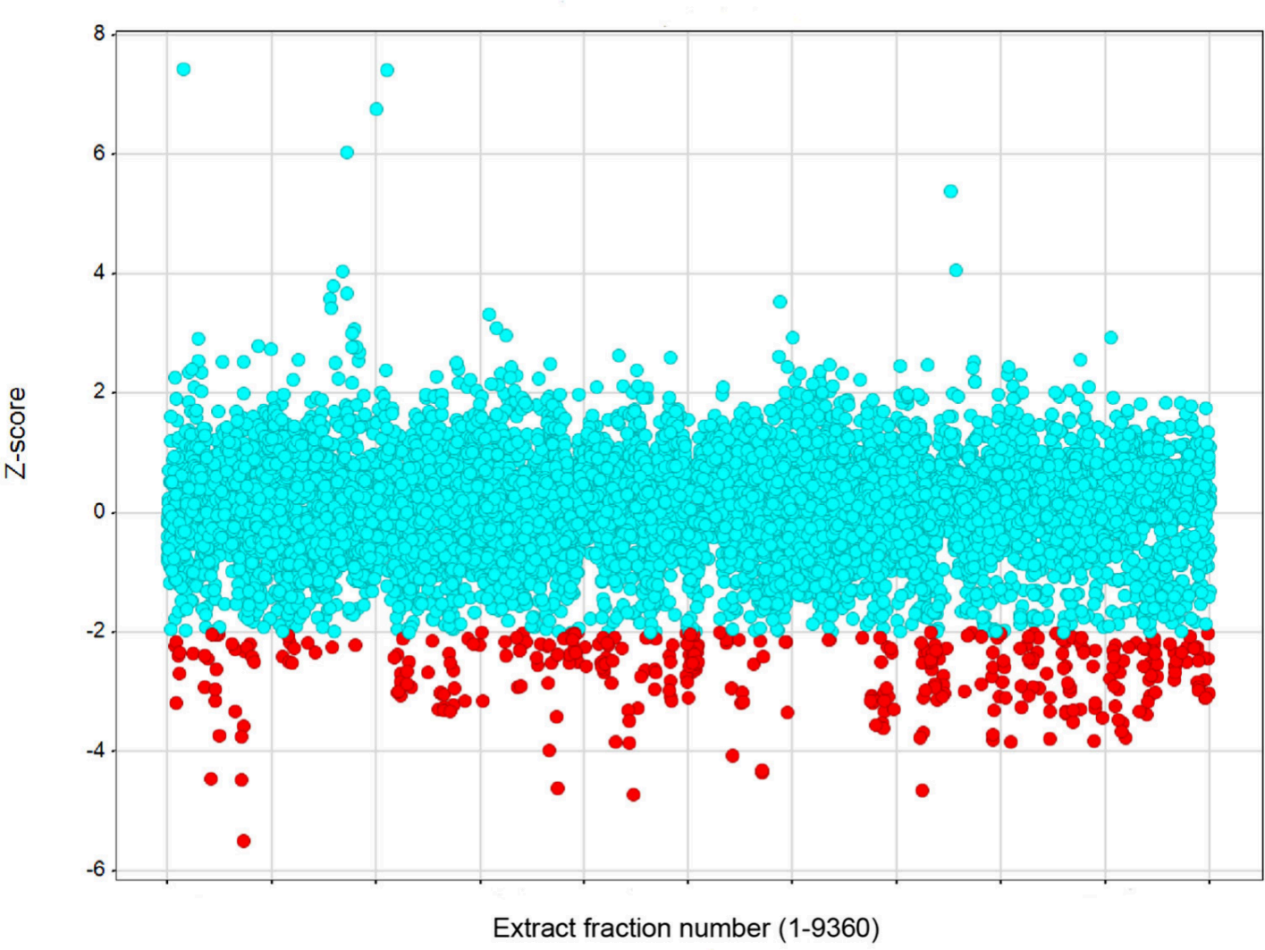
Preliminary Na_v_1.7 screening of 9360 marine extract fractions, expressed as z-scores, which quantify the deviation of each test sample’s fluorescence from the mean fluorescence of the control in the thallium flux assay. A cutoff of z ≤ −2 was applied to designate hits, shown as red dots, while turquoise dots represent fractions with negligible or no activity. The distribution guided the prioritization of promising leads for further investigation.

Some of the most active hits were recorded from midpolar fractions obtained from organisms in the phylum Cnidaria, particularly those belonging to the genera *Sinularia*, *Sarcophyton*, and *Palythoa* (Figure S1, Table S1). Cnidarian venoms, which are often toxic to humans, serve ecological roles in predation and offense and exhibit cytotoxic, hemolytic, and other biological activities with potential therapeutic applications.^30–33^ *Palythoa* spp., zoanthid corals, produce palytoxin, a highly potent natural toxin.^34,35^ Although human cases are rare, palytoxin poisoning can occur following the consumption of contaminated seafood. Other exposure routes include direct contact with the coral or inhalation of dust, vapor, or aerosols in aquarium settings or marine environments.^36,37^ Lacano-Pérez et al.^38^ reported that the venom from *Palythoa caribaeorum* inhibits voltage-gated sodium (Na_v_1.7) and calcium (Ca_v_2.2) channels, suggesting the venom’s potential as a modulator of these ion channels. Beyond the genus *Palythoa*, other cnidarians including *Sinularia* spp. and *Sarcophyton* spp. are known to be a rich source of secondary metabolites, particularly bioactive terpenoids.^39,40^ More than 200 novel compounds have been isolated from various *Sinularia* species, with cembrane terpenoids being among the most commonly reported structural classes.^41–43^ The broad pharmacological potential of *Sinularia* extracts and isolated compounds is well documented in reviews.^39,42,43^

Fractions belonging to *Cribrochalina* sp. and some other sponges (phylum Porifera) were also active in our preliminary screen (Figure S1). In a previous study, 23 linear and branched monoacetylene lipids were isolated from Caribbean *Cribrochalina vasculum*, some of which were selectively cytotoxic.^44^ Two acetylenic alcohols isolated from *Cribrochalina vasculum* inhibited the phosphorylation of IGF-1Rβ and reduced its target signaling molecules IRS-1 and PDK1, indicating their potential in antitumor treatment.^45,46^ Other secondary metabolites reported from *Cribrochalina* spp. include alkaloids, isoquinolines, phosphorylated sterol sulfates, cyclic and linear peptides, and additional acetylenic alcohols.^47–52^ *Gibsmithia hawaiiensis*, a gelatinous red alga, was also among the most active hits. The chemistry and biological activity of this species and its genus remain unexplored, with no prior published reports.

Among the hits in the current study, *Halymenia* sp., an understudied red alga, was selected for further investigation. Over 150 species of the *Halymenia* genus have been described with wide distribution across the Indian and Pacific Oceans.^53^ Previous reports have shown that *Halymenia* sp. releases allelopathic compounds that prevent biofilm formation and interfere with bacterial quorum sensing.^54,55^ Although a report^56^ considered *Halymenia dilatata* as a food source for certain herbivorous marine organisms including sea urchins and some fish species, our field observations revealed relatively intact tissues with little sign of fouling or scarring by herbivores, suggesting it could be chemically defended. In previous studies, the hexane-soluble fraction of *Halymenia durvillei* exhibited moderate cytotoxicity against MDA-MB-231 breast cancer cells.,^57^ while crude methanol extract and fractions of *Halymenia palmata* demonstrated moderate to weak mosquito larvicidal activity.^58^ Although the active constituents were not identified in either study, GC-MS profiling tentatively identified long-chain fatty acids, their esters, and other hydrocarbons in both *H. durvillei* and *H. palmata*. Sulfated polysaccharides from *Halymenia floresii* inhibited herpes simplex virus type 1 *in vitro*, with an EC_50_ of 3.3 μg/mL.^59^ In addition, sulfated galactans isolated from *Halymenia dilatata* showed antibacterial activity against pathogens infecting tilapia.^60^

Following its identification as an active hit in the thallium flux assay (Figure 2A), the dichloromethane-soluble fraction of *Halymenia* sp. was prioritized for chromatographic separation to isolate its bioactive constituents. The extract fraction was observed to be active against Na_v_1.7 and could be a source of potential analgesic leads.^61^

**Figure 2:**
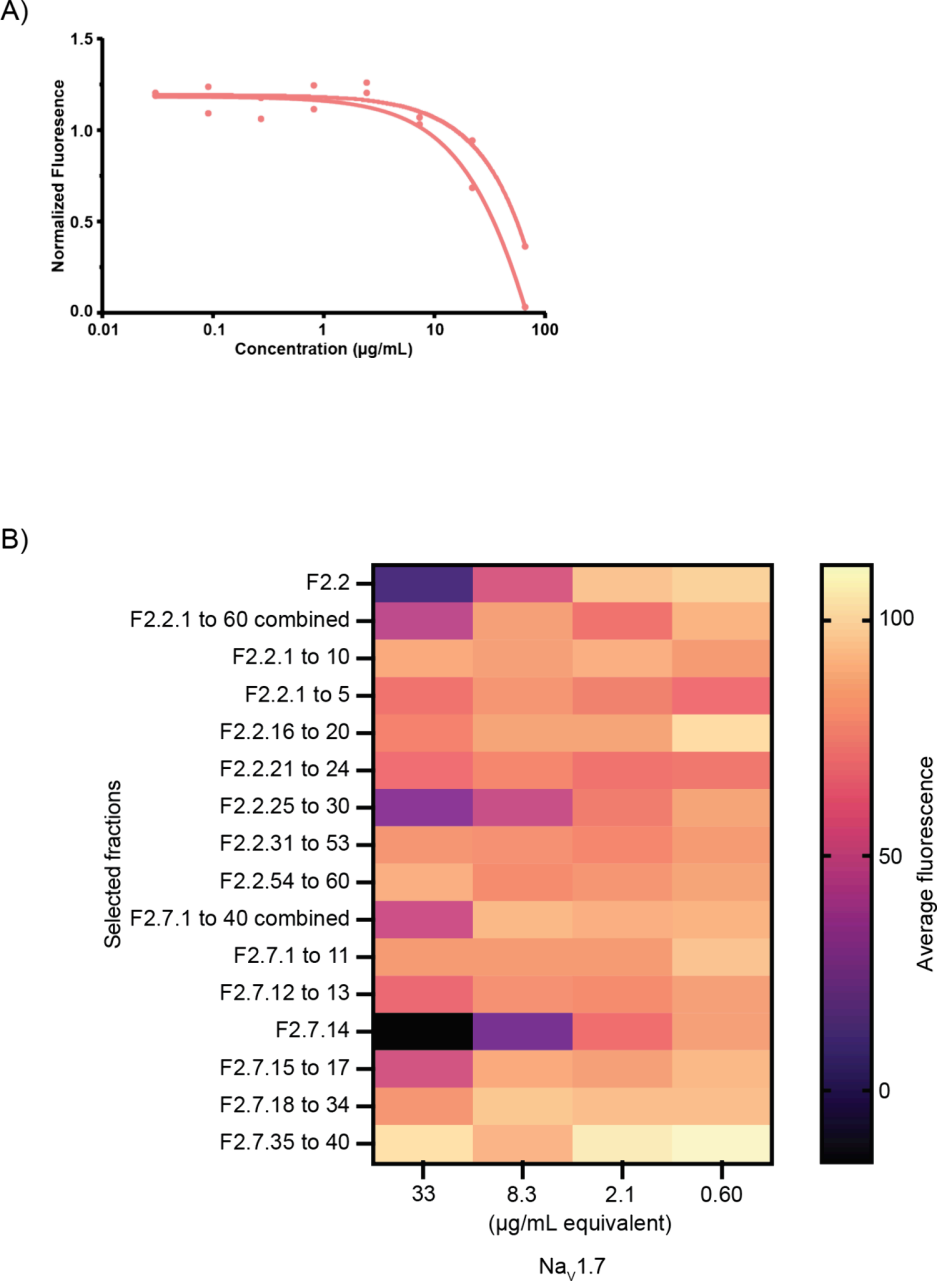
(A) Dose–response curve for two replicates of the dichloromethane-soluble fraction derived from extracts of *Halymenia* sp., tested for inhibition of the Na_v_1.7 channel in the thallium flux assay. Fluorescence was measured to evaluate the electrical conductivity of Na^+^ ions across the channel, with a decrease in normalized sodium flux indicating greater inhibition. The two replicates originated from the same algal collection but were extracted and fractionated separately, highlighting the consistency of bioactivity within the extract, with an average IC_50_ value of 31.7 μg/mL. (B) Primary screening results of the dichloromethane-soluble subfractions of *Halymenia* sp., presented as a heatmap. Darker colors indicate greater inhibition of Na_v_1.7 channel. F2.2.1 to 10 means that combined fractions 1 through 10 were screened for biological activity. Fractions 2.2 and 2.7.14 were measured in μg/mL, whereas others were reported as μg/mL equivalents.

Bean and co-workers^62^ reported that inhibition of Na_v_1.7 channel leads to a decrease in the electrical excitability of primary sensory neurons thereby producing analgesia. Our study employed normalized fluorescence to evaluate the electrical conductivity of sodium channels, with a decrease in fluorescence signifying enhanced inhibition. The assay results revealed a concentration-dependent inhibition of the Na_v_1.7 channel by selected marine extract fractions (Figure 2). The tarantula venom peptide, ProTx-II, known for selectively inhibiting Na_v_1.7 was used in a ^125^I-ProTx-II binding assay previously described by Schmalhofer et al.,^63^ as a validation technique (data not shown).

The dichloromethane-soluble fraction of *Halymenia* sp. was subjected to preparative thin-layer chromatography (prep TLC), yielding subfractions F2.1 to F2.8. F2.2 was further separated by reversed-phase high-performance liquid chromatography (HPLC) to generate F2.2.1 to F2.2.60, while HPLC fractionation of F2.7 produced subfractions F2.7.1 to F2.7.40. The subfractions were combined based on chromatographic profiles and screened in the thallium flux assay (Figure 2B). Among these, subfractions F2.7.14 and F2.2.25 to F2.2.30 exhibited the highest bioactivity, resulting in the isolation of two bioactive glycoglycerolipids, 1-palmitoyl-2-oleoyl-3-(β-D-galactosyl)-glycerol (**1**) and sulfoquinovosyldiacylglycerin (**2**).

Compound **1** was described in a previous study^64^ by our group as a glycosylated glycerolipid, with molecular formula C_43_H_80_O_10_ (*m*/*z* 774.6086 [M+NH_4_]^+^). Most aspects of its molecular structure were elucidated by NMR spectroscopy, mass spectrometry (MS), and microcrystal electron diffraction (MicroED).^64^ At that time, the position of the isolated olefin within the oleoyl chain could not be determined, as three-bond heteronuclear multiple bond correlation (HMBC) signals were only observed for adjacent carbons, with no signals detected for the methyl at C-18’’ or the carbonyl at C-1’’, ruling out its position within three bond lengths of the terminal carbons. Although crystals suitable for MicroED analysis were obtained, disorder within the crystal lattice, likely due to the flexibility and conformation of the lipid chain, prevented precise identification of the positioning of the double bond in that portion of the molecule. In the current study, we turned to a recent innovation in MS to pinpoint the location of the 1,2-disubstituted olefin.^65^ Triboelectric nanogenerators (TENG), which convert mechanical energy to electric current, have been used as an effective power supply (high voltage and low current) for nanoelectrospray ionization (nanoESI). TENG-driven nanoESI ion sources produce pulsed corona discharges that trigger gas phase reactions which aid in structure elucidation.^66,67^ With the aid of TENG-MS, the olefinic site was unambiguously localized in the negative ionization mode using the method described by Fernández and co-workers.^65^ TENG-MS yielded diagnostic fragments at *m*/*z* 155.1076 and 171.1024, corresponding to the ethylene and aldehyde fragments from C-9’’/C-10’’ bond cleavage, affording identification of the site of unsaturation on the MS^3^ spectrum (Figures 4A and S2). An oxidized species of the C18:1 fatty acid fragment was observed at *m*/*z* 297.2434 in the MS^2^ fragment ion spectrum (Figure S3). Adduct ions corresponding to [M+Ac–H]^−^ and [MO+Ac–H]^−^ were observed in the MS^1^ spectrum (Figure S4). Compound **1** was thus established as 1-palmitoyl-2-oleoyl-3-(β-D-galactosyl)-glycerol. Its first isolation was from the roots of *Arisaema amurense* Maxim., Araceae and later from other vascular plants, including *Lycium barbarum* L., Solanceae and *Aralia elata* (Miq.) Seem., Araliaceae, as well as from the brown alga *Sargassum horneri* (Turner) C. Agardh, Sargassaceae.^68–71^ Even though glycoglycerolipids are associated with biological activities such as antitumor,^72,73^ antiviral^74,75^ and anti-inflammatory,^76,77^ Ma et al.^71^ reported that **1** failed to inhibit triglyceride accumulation in 3T3-L1 adipocytes while Jung et al.^68^ observed a weak cytotoxicity against murine leukemia (P388) and human colon adenocarcinoma (DLD-1) cell lines.

The molecular formula of **2** was determined to be C_41_H_78_O_12_S by accurate mass electrospray ionization MS (*m*/*z* 812.5558 [M+NH_4_]^+^). The loss of a palmitoyl group resulted in an MS fragment ion being observed at *m*/*z* 539.2889. A mass fragment ion depicting the aglycone moiety (*m*/*z* 551.5038) was observed and a further loss yielded a fragment ion at *m*/*z* 313.2736, thereby confirming the presence of the second saturated C-16 acyl chain.

For **2**, a large ^1^H NMR signal at δ_H_ 1.23 ppm was indicative of overlapping saturated methylene groups, suggesting the presence of long-chain fatty acyl chains. The chemical shifts and coupling constants observed for the saccharide were consistent with α-d-glucose.^78^ The sulfonate position was assigned based on the observation of the ^1^H NMR signal for C-6’’’ (δ_C_ 54.7 ppm) further upfield. HMBC correlations confirmed the position of attachments of both fatty acid ester chains and sugar to the glycerol. Compound **2** was established as sulfoquinovosyldiacylglycerin and the observed spectroscopic data was consistent with literature (Figure 4B).^78^ Glycoglycerolipid **2** has been previously isolated in its sulfonic acid form and sodium salt from brown and green algae, *Dictyochloris fragrans* and *Caulerpa racemosa*,^79,80^ respectively, and from bacteria in the *Rhizobiaceae* family,^81^ lichen,^82^ and vascular plants.^79,80,83,84^ It selectively inhibited P-selectin’s adhesion to its ligand with an IC_50_ of 5 μM, induced apoptosis in lymphoblastic leukemia MOLT-4 cell lines in a dose-dependent manner, showed anthelmintic activity against *Raillietina* sp. and moderate antiviral activity against HSV-1 and HSV-2 clinical strains.^59,60,85,86^ Prior to the current study, secondary metabolites isolated from red algae of the *Halymenia* genus have been limited to a few sterols, N-acylsphingosines, and sulfated polysaccharides.^59,60,85,86^ This study is the first to report the isolation of glycoglycerolipids from *Halymenia* species.

Pure **1** and **2** were subjected to the thallium flux assay, revealing **1**, with an IC_50_ value of 6.9 μM, as a moderate Na_v_1.7 inhibitor (Figure 3A). Our primary screening measured membrane potential-dependent fluorescence changes caused by ion flux, whereas patch-clamp electrophysiology, considered the gold standard for assessing ion channel activity,^87^ directly measures ion currents. Pure **1** and **2** were thus further evaluated using automated patch-clamp electrophysiology on human Na_v_1.7 (hNa_v_1.7) channel expressed in HEK cells as a secondary screening.^88^ Inhibition was assessed at four concentrations by measuring Na^+^ currents before and after compound application, compared with tetrodotoxin (TTX) as positive control. State-dependent block was evaluated using Qube patch-clamp electrophysiology by measuring inhibition at membrane potentials of −100 mV (tonic block) and −70 mV (inactivated state block). Surprisingly, given its poor activity in the thallium flux assay, **2** demonstrated greater modulation of membrane potentials than **1**, as indicated by a reduction in Na^+^ current across tested concentrations in both states (Figures 3B, S11–S14). The dissociation constants (K_d_), which measure the affinity of a drug for its target receptor, were 49.9 μM for **1** and 25.2 μM for **2** in the inactivated state. Although **2** exhibited a slightly lower K_d_, both compounds demonstrated state-dependent inhibition. Notably, their potency increased against the inactivated state of Na_v_1.7, showing a 5-fold increase compared to the resting state. Additionally, **2** displayed clear dose-dependent activity. This binding behavior aligns with the mechanism observed in local anesthetics such as lidocaine.^89,90^

**Figure 3:**
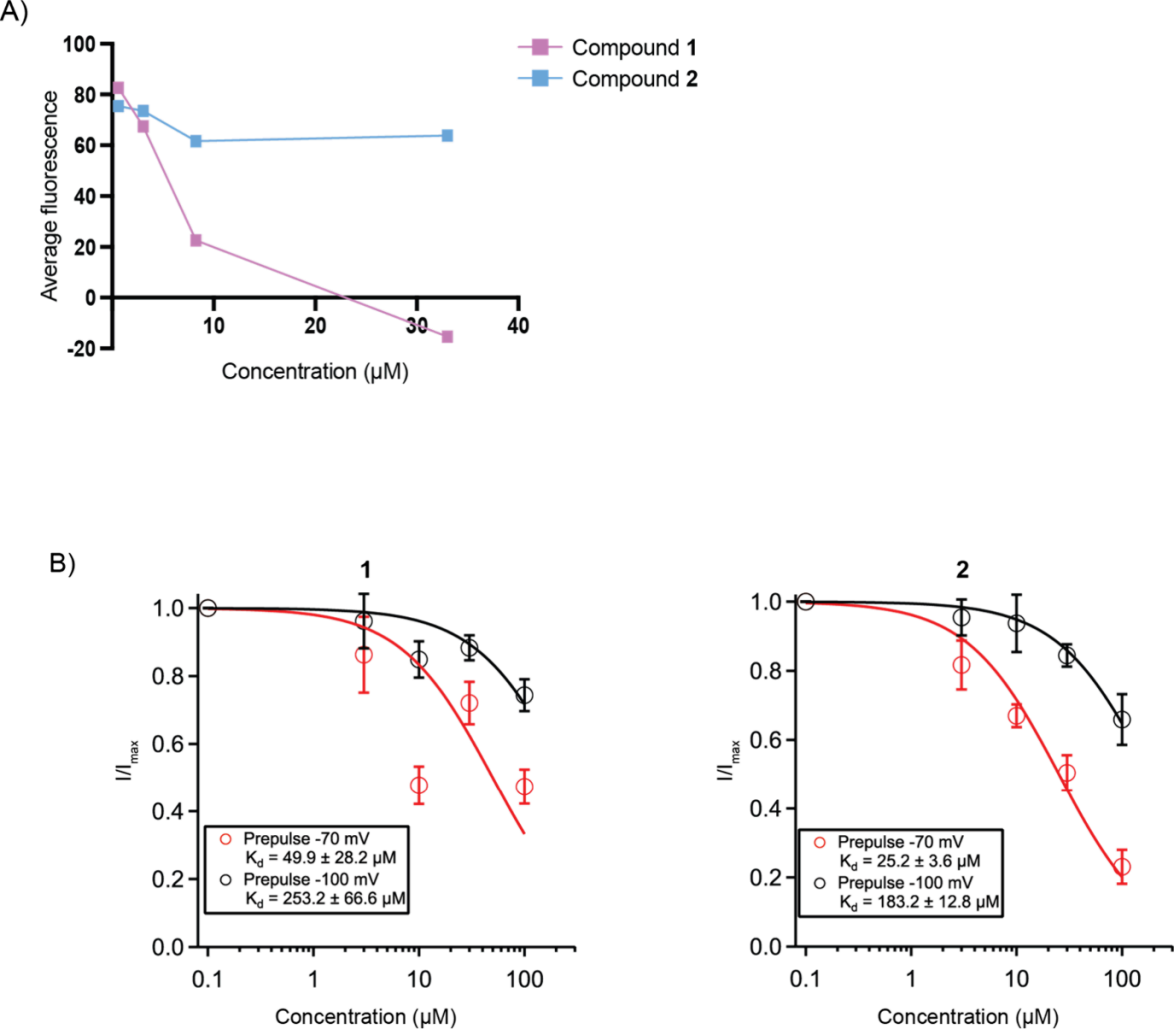
(A) Dose–response curves of **1** and **2** against Na_v_1.7 channel using the thallium flux assay. The *y*-axis represents relative fluorescence as a measure of ion flux. In this assay, **1** exhibited greater potency compared to **2**. (B) Dose–response curves of **1** and **2** against human Na_v_1.7 (hNa_v_1.7) channels using the Qube automated patch-clamp assay, with greater inhibition indicated by a decrease in current (I). *I*_max_ is the control current in 0.1% DMSO before compound addition. The dissociation constant (K_d_) was determined for each compound in the tonic block (−100 mV) and inactivated (−70 mV) states using a modified Hill’s equation.^91^

**Figure 4:**
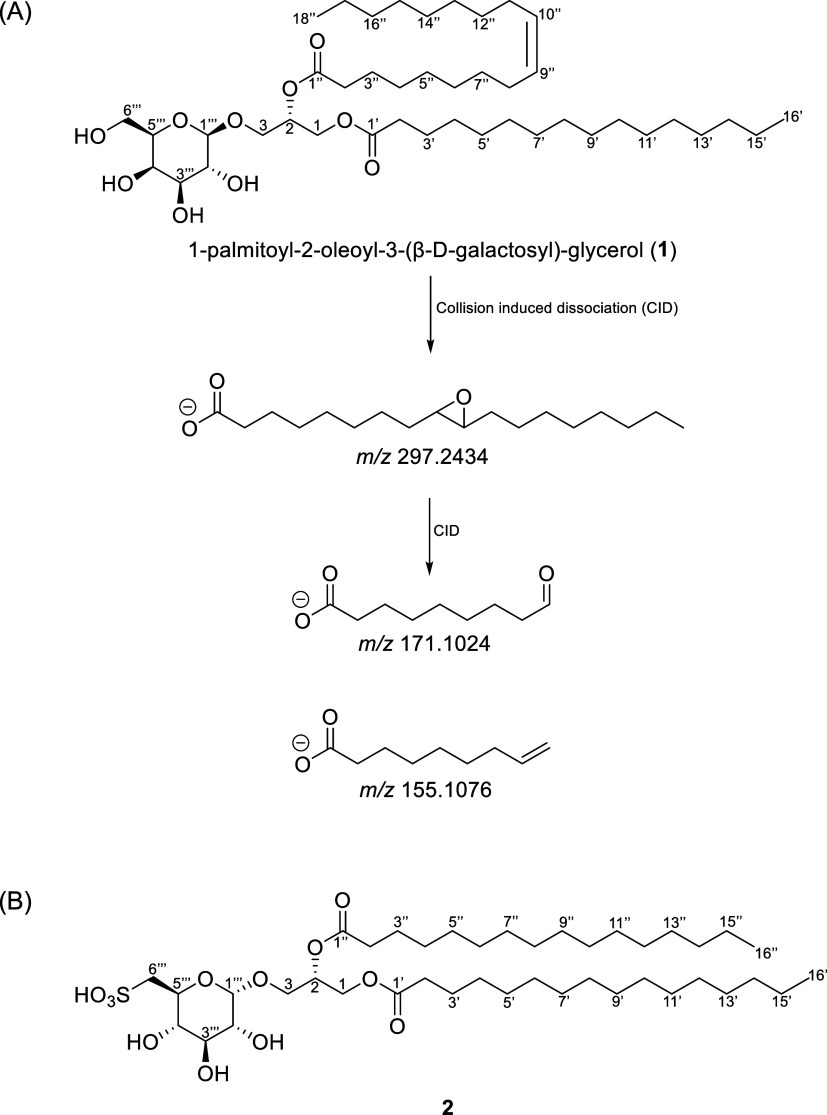
(A) Proposed fragmentation pattern for 1-palmitoyl-2-oleoyl-3-(β-D-galactosyl)-glycerol (**1**) via TENG-MS^n^, revealing the position of the fatty acid olefin at C-9. An epoxide ring is formed during TENG ionization, enabling subsequent fragmentation at the double bond position. (B) Structure of sulfoquinovosyldiacylglycerin (**2**).

Some functional groups of **1** and **2** have been previously found pharmacologically relevant in studies related to pain management. For instance, transient receptor potential vanilloid 1 (TPRV1), a nonselective cation channel associated with pain, itch, and inflammation, was inhibited by oleic acid, a naturally occurring omega-9 unsaturated fatty acid, resulting in reduced pain and itch in mice.^92^ Polysorbate 80, a nonionic emulsifier derived from oleic acid and polyethoxylated sorbitan, was also shown by Kim and Choi^93^ in their patch-clamp study to inhibit action potential generation in neurons and block Na_v_1.7 in a concentration-dependent manner. Notably, **1** contains an oleoyl chain, which may contribute to the activity observed in the preliminary screening.

When screening a library of known synthetic selective Na_v_1.7 inhibitors, aryl sulfonamides, McKerrall et al.^94^ reported that a one-unit increase in lipophilicity led to a 75-fold increase in Na_v_1.7 inhibition. Similarly, some lipid-soluble neurotoxins, such as brevetoxins, ciguatoxins, veratridine, and aconitine, exert their primary pharmacological effect by targeting voltage-gated sodium channels through agonistic, partial agonist, and allosteric mechanisms.^95,96^ Although glycoglycerolipids are structurally distinct from these modulators, their amphipathic nature may contribute to voltage-gated sodium channel modulation.

In conclusion, our screening of a large marine extract library led to the identification of extract fractions from diverse taxa, including red algae, cnidarians, and sponges, that inhibit the voltage-gated sodium channel Na_v_1.7. This highlights the broad phylogenetic range of marine organisms as valuable sources of novel sodium channel modulators. Isolated natural products from one of the active hits showed preliminary pharmacological activity that presents an opportunity for further exploration. These findings underscore the value of marine biodiversity in the search for novel therapeutics and support continued exploration of marine-derived compounds in drug discovery.

## Experimental Section

### General Experimental Procedures

Optical rotatory dispersion measurements were performed on a Jasco DIP-360 digital polarimeter using methanol as the solvent. NMR spectra were acquired in DMSO-*d*_6_ on an 18.8 T Bruker Avance IIIHD spectrometer with a 3 mm triple resonance broadband cryoprobe. Chemical shifts were reported in parts per million relative to the solvent residual peaks δ_H_ 2.50 and δ_C_ 39.52. NMR spectroscopic data were processed and analyzed using MestReNova 11.0.4. High-resolution mass spectrometry (MS) and TENG-MS were performed using a ThermoFisher Orbitrap ID-X instrument. Preparative thin layer chromatography (TLC) separations were performed using Silicycle (20 × 20 cm, 200 μm thickness) TLC plates. High-performance liquid chromatography (HPLC) separations were conducted on a Waters 1525 binary pump equipped with a Waters 2996 photodiode array detector and an Altech 800 evaporative light scattering detector.

### Specimen Collection and Identification

Marine organisms were collected as part of an NIH ICBG program in Fiji and the Solomon Islands over a 12-year period (2006–2018). Each specimen was extracted three times using methanol, followed by vacuum liquid chromatography (VLC) fractionation using HP20SS Diaion resin at a dry extract-to-resin ratio of 1:20. The crude extract was adsorbed onto the resin, washed with water to remove salts, and sequentially eluted using 50% aqueous methanol (Fraction A), 80% aqueous methanol (Fraction B), methanol (Fraction C), and acetone (Fraction D), yielding four extract fractions.^97^ The midpolar fractions (B and C) were selected to form the extract library for further studies. Each extract fraction was prepared at volumes of 100, 300, and 600 nL, plated into a 384-well plate, and screened using the thallium flux assay.

*Halymenia* sp. (sample G-0815) was collected from Mango Bay locale, Vitu Levu Island, Fiji Islands (S 18°14.184’, E 177°46.86′) in April 2010 at depths ranging from 5 to 40 m. It had a soft texture with mucus and was pink-red in color. The collection was identified as an uncertain species of *Halymenia* based on the morphological features and comparison to the previous description.^98^ Voucher specimen and morphological samples were preserved in formaldehyde and ethanol, respectively, at the Georgia Institute of Technology and at the University of the South Pacific (USP) in Suva, Fiji. The bulk sample was stored in a −80 °C freezer.

On the day of collection, in the field, 20 g wet weight of *Halymenia* sp. was extracted with methanol, followed by fractionation using Diaion HP-20SS as stationary phase once extracts were returned to the lab at USP. Elution was carried out sequentially using 50% aqueous methanol, 80% aqueous methanol, methanol and acetone, resulting in four extract fractions. These fractions were screened against a human embryonic kidney (HEK) cell line expressing Na_v_1.7 to identify bioactive fractions.

Separately and at a later date, additional material from the same collection of *Halymenia* sp. (428.2 g wet weight) was extracted exhaustively using MeOH, MeOH/CH_2_Cl_2_ (1:1) and CH_2_Cl_2_, resulting in 17.4 g of combined extract after drying *in vacuo*. The crude extract was suspended in 10% aqueous methanol and partitioned with hexanes to afford the hexanes-soluble fraction (F1). The aqueous portion was adjusted to 40% aqueous methanol and partitioned with CH_2_Cl_2_, resulting in the CH_2_Cl_2_-soluble fraction (F2). MeOH was evaporated from the aqueous phase and the residual aqueous portion was partitioned with ethyl acetate (EtOAc), yielding the EtOAc-soluble fraction (F3). The remaining H_2_O-soluble fraction was designated as F4.

The CH_2_Cl_2_-soluble fraction (0.7 g) was subjected to silica gel Prep TLC in multiple batches and eluted with MeOH/EtOAc (1:4), affording 8 fractions (F2.1 – F2.8). Fraction F2.7 (15.4 mg) was subjected to HPLC separation on a normal phase silica column (5 μm, 4.6 × 250 mm) using ethyl acetate to MeOH/EtOAc (3:2) over 20 min (solvent flow rate 1 mL/min). This resulted in 40 fractions (F2.7.1 – F2.7.40) that were collected at 30-s intervals. F2.7.14 contained pure **1** (1.5 mg) which was quantified with quantitative NMR spectroscopy using a capillary filled with benzene-*d*_6_ as an internal standard and calibrated against caffeine.^99^ It also afforded crystals suitable for MicroED analysis,^64^ a portion of which was used for bioassay. An adjacent HPLC fraction, F2.7.15, also consisted of pure **1** that was used for NMR, HRMS, and TENG-MS analysis. The partial structural elucidation of **1**, including its NMR and HRMS data, was documented in our previous study.^64^

Fraction F2.2 was separated on the HPLC using a reversed phase C_18_ silica column (5 μm, 4.6 × 250 mm) using a solvent gradient of 1:1 MeOH/H_2_O to 1:9 CH_3_CN/IPA over 15 min, held for 7 min at 1:9 CH_3_CN/IPA, followed with 1:9 CH_3_CN/IPA to 1:1 MeOH/H_2_O over 3 min and isocratic 1:1 MeOH/H_2_O for 5 min (solvent flow rate 0.9 mL/min). 60 fractions (F2.2.1 – F2.2.60) were generated with F2.2.25 – F2.2.30 containing pure **2** (1.7 mg).

#### Sulfoquinovosyldiacylglycerin (**2**)

Yellow solid; [α]^25^_D_ + 23 (*c* 0.14, MeOH); ^1^H and ^13^C data, Table S2; HRMS *m*/*z* [M+NH_4_]^+^ calcd for C_41_H_78_O_12_S 812.5557, found 812.5558.

### TENG-MS Analysis

1-palmitoyl-2-oleoyl-3-(β-D-galactosyl)-glycerol (**1**) (250 μM) was solubilized in DMSO and diluted into 6:1:1 acetone/H_2_0/MeOH with 200 mM ammonium acetate, a solution which enhances oxidation during TENG ionization. The diluted sample was introduced into the TENG ion source through nanoemitter glass tips pulled in-house using borosilicate thin-wall glass capillaries. Additional details can be found elsewhere.^65^

### Thallium Flux Assay

A day before screening, each HEK cell line (in duplicate) is plated in a 384-well plate (Greiner 781090) at a density of 20,000 cells/well and grown for ∼24 h in 37 °C incubator. We used DMEM+10%FBS+P/S with selective antibiotics for subtype specific Na_v_ expressing cell lines. On the day of screening, one compound plate (Greiner 784201) was filled with 30 μL of Live Cell Imaging Solution containing 140 mM NaCl, 2.5 mM KCl, 1.8 mM CaCl_2_, 1.0 mM MgCl_2_, 20 mM HEPES pH 7.4 (A59688DJ, Thermo Fisher). Next, 300 nL of small molecules were pin-transferred to each compound plate. Cell plates were taken out of the incubator and media was removed and washed with 20 μL of Buffer twice using Bravo liquid handler (Agilent), and 20 μL of FluxORII Green dye (F20017, Thermo Fisher Scientific) was added to each well. Cell plates were incubated at RT for 30 min in the dark. After incubation, 10 μL of compounds from the compound plate was added to the HEK cells for 15 min at RT in the dark (one compound plate per two cell plates).

Stimulus buffer composed of KHCO_3_, KCl, CaSO_4_, MgSO_4_, Glucose, HEPES, TiSO_4_ was added to the assay plates and read using the FDSS7000EX (Hamamatsu Photonics) where 10 μL of Stimulus buffer is added to each well at the appropriated depth and speed of 15s/μL, making a final volume of 40 μL. Continuous readings (Ex480:Em540) were taken at .23s intervals in the wells. Raw data is normalized by background signal of each well. The initial slopes were calculated within 10 s time zone 3 s after the addition of stimulus. Z-scores were then calculated based on plate average of experimental wells. A compound is considered a hit if the z-score is lower than −2. Both replicates must meet criteria to be considered a hit.

### Human Na_v_1.7 Cell Line

A stable line of HEK293 cells expressing human Na_v_1.7 voltage-gated sodium channel alpha subunit (1) were maintained in EMEM media (Corning) containing 10% FBS, 1% penicillin/streptomycin (Sigma), and 700 μg/mL Geneticin (Sigma) under 5% CO_2_ in a 37 °C incubator.

### Cell Preparation for Electrophysiology

Cells were cultured to approximately 70% confluency to maximize cell viability and channel expression. On the day of recording, cells were washed twice with divalent-free phosphate-buffered saline (Corning) and incubated in Detachin enzyme solution (Genlantis) for 3 min at 37 °C. The cell enzyme mixture was diluted 1:10 in serum-free EX-CELL media (Sigma) and centrifuged at 65 G for 2 min. The cell pellet was resuspended in serum-free EX-Cell media (Sigma), and cell density and viability were measured using a Countless 3 cell counter (Invitrogen). Volume was adjusted to a cell density was 1.5 – 2 × 10^6^ cells/mL with cell viability ≥ 98%.

### Automated Patch-Clamp Electrophysiology

Voltage clamp recordings of Na_v_1.7 were measured using a Qube 384 automated patch-clamp system (Sophion Biosciences). All recordings were performed using multihole recording chips that permit simultaneous recording of up to ten cells in the same well. The currents from all cells within a well were summed to form the macroscopic Na^+^ current, and the leak generated from patch holes without cells was measured using 10 mV hyperpolarizing pulses and subtracted accordingly. The extracellular recording solution contained in mM: 145 NaCl, 4 KCl, 2 CaCl_2_, 1 MgCl_2_, 10 HEPES, and 10 glucose, with pH adjusted to 7.4 using NaOH and osmolarity between 300 and 305 mOsm/L. The intracellular solution contained in mM: 140 CsF, 10 NaCl, 1 EGTA, 10 HEPES, and 10 glucose with pH adjusted to 7.2 with CsOH and osmolarity of 320 mOsm/L. All compounds were prepared in the same extracellular solution with the addition of 0.1% Pluronic-F68 (Sigma), a surfactant poloxamer that enhances solubility of lipophilic compounds. All recordings were performed with temperature control at 25 °C.

Data acquisition and operation of the Qube was controlled with Sophion Viewpoint software (v9.0.42). Whole-cell recordings were obtained using the “Sophion Standard” protocol, in which cells were captured on each patch hole with a negative suction pressure of −80 mbar. Once the patch hole resistance increased, indicating presence of a cell, the pressure was switched to −10 mbar for holding and seal formation. After gigaseal formation of ≥ 800 MΩ, whole-cell configuration was achieved by applying two negative pressure pulses from −10 mbar to −250 mbar for two seconds, then to −350 mbar for another two seconds. All cells were held at a membrane potential of −100 mV, and sodium currents were evoked using protocols designed to evaluate state-dependent interactions between the channel and test compounds. Currents were digitized and filtered at 50 kHz with an eighth-order Bessel filter and a corner frequency of 16.66 kHz. After data acquisition, the current and voltage command waveforms were exported from Sophion Analyzer software (v9.0.42) and imported into Igor Pro (version 6.7, WaveMetrics) for analysis.

### Data Analysis

Raw current traces were corrected for linear capacitative and leak currents, which were determined using 10 mV hyperpolarizing steps applied from resting potential of −100 mV and both scaled and subtracted accordingly. Maximum inward Na^+^ currents were measured using 10 ms test pulses to +10 mV. To measure tonic block of resting state channels, cells were held at −100 mV membrane potential prior to the test pulse. Inactivated state-dependent block was evaluated in two ways: first, cells were held at −70 mV for 2.5 s before test pulse; second, cells were held at −60 mV for 2.5 s, then hyperpolarized to −100 mV for 40 ms before the test pulse. The former approach allows measurement of channel block at the approximate half maximal voltage (*V*_1/2_) for Na_v_1.7, while the latter permits drug exposure to a greater fraction of inactivated channels but requires a hyperpolarization period before the test pulse to allow some recovery from fast inactivation. Channel block was determined by calculating the ratio of inward Na^+^ current after drug exposure to the current at the end of the control period. N values for each group include wells with currents ≥ 3 nA, and error bars represent standard deviation (SD). Dose–response analysis was used to estimate the dissociation constant (K_d_) for each compound using the fit to a Hill-Langmuir equation equation modifed to account for a nonzero baseline, I= I_Min_+(I_Max_-I_Min_)/(1+[Drug]/K_d_), where I is the Na^+^ current following 7 min of drug exposure at a concentration [Drug], I_Max_ is the control current in 0.1% DMSO before drug addition, and I_Min_ is a baseline to account for a small remaining current in saturating drug concentrations. All data analyses were completed in Igor Pro (v6.7).

## Supplementary Material



## Notes

Photographs of selected marine organisms with biologically active extracts and their collection sites, NMR and HRMS spectra of isolated compounds, voltage protocol, and primary data from the automated patch-clamp electrophysiology study (PDF)

## References

[1] Goyal S., Goyal S., Goins A. E., Alles S. R. A. (2023). Plant-derived
natural products targeting ion channels for pain. Neurobiology of Pain.

[2] de
Lera Ruiz M., Kraus R. L. (2015). Voltage-Gated Sodium Channels: Structure,
Function, Pharmacology, and Clinical Indications. J. Med. Chem..

[3] Catterall W. A. (1993). Structure
and function of voltage-gated ion channels. Trends in Neurosciences.

[4] Wood J. N., Boorman J. P., Okuse K., Baker M. D. (2004). Voltage-gated sodium
channels and pain pathways. Journal of Neurobiology.

[5] Bagal S. K., Marron B. E., Owen R. M., Storer R. I., Swain N. A. (2015). Voltage
gated sodium channels as drug discovery targets. Channels.

[6] Newman D. J., Cragg G. M. (2020). Natural Products as Sources of New
Drugs over the Nearly
Four Decades from 01/1981 to 09/2019. J. Nat.
Prod..

[7] Chen R., Liu Y., Qian L., Yi M., Yin H., Wang S., Xiang B. (2025). Sodium channels as a new target for
pain treatment. Frontiers in Pharmacology.

[8] McDermott L. A., Weir G. A., Themistocleous A. C., Segerdahl A. R., Blesneac I., Baskozos G., Clark A. J., Millar V., Peck L. J., Ebner D. (2019). Defining
the Functional
Role of Na_v_1.7 in Human Nociception. Neuron.

[9] Renganathan M., Cummins T. R., Waxman S. G. (2001). Contribution of
Nav1.8 Sodium Channels
to Action Potential Electrogenesis in DRG Neurons. Journal of Neurophysiology.

[10] Cai S., Bellampalli S. S., Yu J., Li W., Ji Y., Wijeratne E. M. K., Dorame A., Luo S., Shan Z., Khanna M. (2019). (−)-Hardwickiic Acid and Hautriwaic Acid Induce
Antinociception via Blockade of Tetrodotoxin-Sensitive Voltage-Dependent
Sodium Channels. ACS Chem. Neurosci..

[11] Zhou Y., Cai S., Moutal A., Yu J., Gómez K., Madura C. L., Shan Z., Pham N. Y. N., Serafini M. J., Dorame A. (2019). The Natural Flavonoid Naringenin Elicits Analgesia
through Inhibition of NaV1.8 Voltage-Gated Sodium Channels. ACS Chem. Neurosci..

[12] Wu G., Li L., Chen B., Chen C., Luo D., He B. (2018). Natural meroterpenoids
isolated from the plant pathogenic fungus Verticillium albo-atrum
with noteworthy modification action against voltage-gated sodium channels
of central neurons of Helicoverpa armigera. Pestic. Biochem. Physiol..

[13] Zhou Y., Cai S., Gomez K., Wijeratne E. M. K., Ji Y., Bellampalli S. S., Luo S., Moutal A., Gunatilaka A. A. L., Khanna R. (2020). 1-O-Acetylgeopyxin
A, a derivative of a fungal metabolite, blocks tetrodotoxin-sensitive
voltage-gated sodium, calcium channels and neuronal excitability which
correlates with inhibition of neuropathic pain. Molecular Brain.

[14] Adams D. J., Alewood P. F., Craik D. J., Drinkwater R. D., Lewis R. J. (1999). Conotoxins and their potential pharmaceutical applications. Drug Dev. Res..

[15] Pope J. E., Deer T. R. (2013). Ziconotide: a clinical
update and pharmacologic review. Expert Opinion
on Pharmacotherapy.

[16] Baj A., Bistoletti M., Bosi A., Moro E., Giaroni C., Crema F. (2019). Marine Toxins and Nociception: Potential Therapeutic Use in the Treatment
of Visceral Pain Associated with Gastrointestinal Disorders. Toxins.

[17] Kolosov A., Goodchild C. S., Cooke I. (2010). CNSB004 (Leconotide) Causes Antihyperalgesia
Without Side Effects When Given Intravenously: A Comparison with Ziconotide
in a Rat Model of Diabetic Neuropathic Pain. Pain Medicine.

[18] Duran P., Loya-López S., Ran D., Tang C., Calderon-Rivera A., Gomez K., Stratton H. J., Huang S., Xu Y.-m., Wijeratne E. M. K. (2023). The natural product argentatin C attenuates
postoperative pain via inhibition of voltage-gated sodium and T-type
voltage-gated calcium channels. Br. J. Pharmacol..

[19] Jean Y.-H., Chen W.-F., Sung C.-S., Duh C.-Y., Huang S.-Y., Lin C.-S., Tai M.-H., Tzeng S.-F., Wen Z.-H. (2009). Capnellene,
a natural marine compound derived from soft coral, attenuates chronic
constriction injury-induced neuropathic pain in rats. Br. J. Pharmacol..

[20] Calderon-Rivera A., Loya-Lopez S., Gomez K., Khanna R. (2022). Plant and fungi derived
analgesic natural products targeting voltage-gated sodium and calcium
channels. Channels.

[21] Witkop, B. ; Gössinger, E. Amphibian Alkaloids. In The Alkaloids: Chemistry and Pharmacology, Brossi, A. , Ed.; Vol. 21; Academic Press, 1983; pp 139–253.

[22] McIntosh J. M., Hasson A., Spira M. E., Gray W. R., Li W., Marsh M., Hillyard D. R., Olivera B. M. (1995). A New Family of
Conotoxins That Blocks Voltage-gated Sodium Channels (*). J. Biol. Chem..

[23] Llewellyn L. E. (2006). Saxitoxin,
a toxic marine natural product that targets a multitude of receptors. Natural Product Reports.

[24] McMahon K. L., Vetter I., Schroeder C. I. (2024). Voltage-Gated
Sodium Channel Inhibition
by μ-Conotoxins. Toxins.

[25] Li H.-L., Hadid D., Ragsdale D. S. (2002). The Batrachotoxin
Receptor on the
Voltage-Gated Sodium Channel is Guarded by the Channel Activation
Gate. Mol. Pharmacol..

[26] Grishchenko I. I., Naumov A. P., Zubov A. N. (1983). Gating
and selectivity of aconitine-modified
sodium channels in neuroblastoma cells. Neuroscience.

[27] Jiang H., Zhang Y., Zhang Y., Wang X., Meng X. (2022). An Updated
Meta-Analysis Based on the Preclinical Evidence of Mechanism of Aconitine-Induced
Cardiotoxicity. Frontiers in Pharmacology.

[28] Gulsevin A., Glazer A. M., Shields T., Kroncke B. M., Roden D. M., Meiler J. (2022). Veratridine Can Bind
to a Site at the Mouth of the
Channel Pore at Human Cardiac Sodium Channel NaV1.5. International Journal of Molecular Sciences.

[29] Du Y., Days E., Romaine I., Abney K. K., Kaufmann K., Sulikowski G., Stauffer S., Lindsley C. W., Weaver C. D. (2015). Development
and Validation of a Thallium Flux-Based Functional Assay for the Sodium
Channel NaV1.7 and Its Utility for Lead Discovery and Compound Profiling. ACS Chem. Neurosci..

[30] D’Ambra I., Lauritano C. (2020). A Review of
Toxins from Cnidaria. Marine Drugs.

[31] Lazcano-Pérez F., Zavala-Moreno A., Rufino-González Y., Ponce-Macotela M., García-Arredondo A., Cuevas-Cruz M., Gómez-Manzo S., Marcial-Quino J., Arreguín-Lozano B., Arreguín-Espinosa R. (2018). Hemolytic, anticancer and antigiardial
activity of Palythoa caribaeorum venom. Journal
of Venomous Animals and Toxins including Tropical Diseases.

[32] Mariottini G. L., Pane L. (2014). Cytotoxic and Cytolytic
Cnidarian Venoms. A Review on Health Implications
and Possible Therapeutic Applications. Toxins.

[33] Šuput D. (2009). In vivo effects
of cnidarian toxins and venoms. Toxicon.

[34] Hammond H. L., Roy C. J. (2024). History and Toxinology of Palytoxins. Toxins.

[35] Gleibs S., Mebs D. (1999). Distribution and sequestration of palytoxin in coral reef animals. Toxicon.

[36] Hoffmann K., Hermanns-Clausen M., Buhl C., Büchler M. W., Schemmer P., Mebs D., Kauferstein S. (2008). A case of
palytoxin poisoning due to contact with zoanthid corals through a
skin injury. Toxicon.

[37] Deeds J. R., Schwartz M. D. (2010). Human risk associated
with palytoxin exposure. Toxicon.

[38] Lazcano-Pérez F., Castro H., Arenas I., García D. E., González-Muñoz R., Arreguín-Espinosa R. (2016). Activity of
Palythoa caribaeorum Venom on Voltage-Gated Ion Channels in Mammalian
Superior Cervical Ganglion Neurons. Toxins.

[39] Sheu J.-H., Peng B.-R., Fang L.-S., Hwang T.-L., Su J.-H., Wu Y.-C., Sung P.-J. (2019). Hydroperoxyditerpenoids
from Octocorals. Isr. J. Chem..

[40] Ishigami S., Nakagawa R., Yagi F., Takada H., Suzuki A., Kamada T., Nimura K., Oshima I., Phan C.-S., Ishii T. (2025). Anti-biofouling marine
diterpenoids from Okinawan soft corals. Biofouling.

[41] Yan X., Liu J., Leng X., Ouyang H. (2021). Chemical Diversity and Biological
Activity of Secondary Metabolites from Soft Coral Genus Sinularia
since 2013. Marine Drugs.

[42] Chen W.-t., Li Y., Guo Y.-w. (2012). Terpenoids
of Sinularia soft corals: chemistry and
bioactivity. Acta Pharmaceutica Sinica B.

[43] Rodrigues I. G., Miguel M. G., Mnif W. (2019). A Brief Review on New Naturally Occurring
Cembranoid Diterpene Derivatives from the Soft Corals of the Genera
Sarcophyton, Sinularia, and Lobophytum Since 2016. Molecules.

[44] Kovalerchik D., Zovko A., Hååg P., Sierakowiak A., Viktorsson K., Lewensohn R., Ilan M., Carmeli S. (2022). Cytotoxic
Alkylynols of the Sponge Cribrochalina vasculum: Structure, Synthetic
Analogs and SAR Studies. Marine Drugs.

[45] Zovko A., Novak M., Hååg P., Kovalerchick D., Holmlund T., Färnegårdh K., Ilan M., Carmeli S., Lewensohn R., Viktorsson K. (2016). Compounds
from the marine sponge Cribrochalina vasculum offer a way to target
IGF-1R mediated signaling in tumor cells. Oncotarget.

[46] Zovko A., Viktorsson K., Hååg P., Kovalerchick D., Färnegårdh K., Alimonti A., Ilan M., Carmeli S., Lewensohn R. (2014). Marine Sponge
Cribrochalina vasculum
Compounds Activate Intrinsic Apoptotic Signaling and Inhibit Growth
Factor Signaling Cascades in Non-Small Cell Lung Carcinoma. Molecular Cancer Therapeutics.

[47] Hallock Y. F., Cardellina J. H., Balaschak M. S., Alexander M. R., Prather T. R., Shoemaker R. H., Boyd M. R. (1995). Antitumor Activity
and Stereochemistry of Acetylenic Alcohols from the Sponge Cribrochalina
vasculum. J. Nat. Prod..

[48] Dai J.-R., Hallock Y. F., Cardellina J. H., Boyd M. R. (1996). Vasculyne, a New
Cytotoxic Acetylenic Alcohol from the Marine Sponge Cribrochalina
vasculum. J. Nat. Prod..

[49] Fujita M., Nakao Y., Matsunaga S., Seiki M., Itoh Y., van Soest R. W. M., Heubes M., Faulkner D. J., Fusetani N. (2001). Isolation
and structure elucidation of two phosphorylated sterol sulfates, MT1-MMP
inhibitors from a marine sponge Cribrochalina sp.: revision of the
structures of haplosamates A and B. Tetrahedron.

[50] Kariya Y., Kubota T., Fromont J., Kobayashi J. i. (2006). Pyrinadines
B-G, new bis-pyridine alkaloids with an azoxy moiety from sponge Cribrochalina
sp. Bioorg. Med. Chem..

[51] Plubrukarn A., Yuenyongsawad S., Thammasaroj T., Jitsue A. (2003). Cytotoxic Isoquinoline
Quinones from the Thai Sponge Cribrochalina. Pharmaceutical Biology.

[52] Urda C., Pérez M., Rodríguez J., Jiménez C., Cuevas C., Fernández R. (2016). Pembamide,
a N-methylated linear
peptide from a sponge Cribrochalina sp. Tetrahedron
Lett..

[53] Manohong P., Sornkaew N., Meemon K., Chumphoochai K., Sobhon P., Tamtin M., Sichaem J., Mingvanish W., Srisuwannaket C., Mingvanish W., Niamnont N. (2021). Isolation of 3-(Hydroxyacetyl)
indole and Indole-3-carboxylic acid from Red Alga Halymenia durvillei:
Their anti-lung cancer cell and in vivo anti-aging activity. Asian J. Chem..

[54] A
Abdul Malik S., Saha M., Taupin L., Bedoux G., Bourgougnon N., Robledo D. (2022). Identification of the quorum sensing
signal of the opportunistic pathogen inducing bleaching disease in
the red macroalga Halymenia floresii holobiont. Applied Phycology.

[55] A
Abdul Malik S., Bedoux G., Robledo D., García-Maldonado J. Q., Freile-Pelegrín Y., Bourgougnon N. (2020). Chemical defense
against microfouling by allelopathic active metabolites of Halymenia
floresii (Rhodophyta). Journal of Applied Phycology.

[56] Hussni
Hasan N. R., Yogarajalakshmi P., Vasantha-Srinivasan P., Shehata W. F., Radhakrishnan N., Jayakodi S., Karthi S., Senthil-Nathan S., Hamed Mansour H. E., Ghazzawy H. S. (2023). From
the Sea to Mosquito Control: The Potential of Halymenia dilatata Marine
Alga as an Eco-Friendly Mosquitocidal Agent. Sustainability.

[57] Sangpairoj K., Settacomkul R., Siangcham T., Meemon K., Niamnont N., Sornkaew N., Tamtin M., Sobhon P., Vivithanaporn P. (2022). Hexadecanoic
acid-enriched extract of Halymenia durvillei induces apoptotic and
autophagic death of human triple-negative breast cancer cells by upregulating
ER stress. Asian Pacific journal of tropical
biomedicine.

[58] Deepak P., Balamuralikrishnan B., Park S., Sowmiya R., Balasubramani G., Aiswarya D., Amutha V., Perumal P. (2019). Phytochemical
profiling
of marine red alga, Halymenia palmata and its bio-control effects
against Dengue Vector, Aedes aegypti. South
African Journal of Botany.

[59] Pliego-Cortés H., Hardouin K., Bedoux G., Marty C., Cérantola S., Freile-Pelegrín Y., Robledo D., Bourgougnon N. (2022). Sulfated Polysaccharides
from Seaweed Strandings as Renewable Source for Potential Antivirals
against Herpes simplex Virus 1. Marine Drugs.

[60] Vinosha M., Palanisamy S., Anjali R., Li C., Yelithao K., Marudhupandi T., Tabarsa M., You S., Prabhu N. M. (2020). Sulfated
galactan from Halymenia dilatata enhance the antioxidant properties
and prevents Aeromonas hydrophila infection in tilapia fish: In vitro
and in vivo study. Int. J. Biol. Macromol..

[61] Erdemli G., Kim A. M., Ju H., Springer C., Penland R. C., Hoffmann P. K. (2012). Cardiac Safety Implications
of hNav1.5 Blockade and
a Framework for Pre-Clinical Evaluation. Frontiers
in Pharmacology.

[62] Lee S., Jo S., Talbot S., Zhang H.-X. B., Kotoda M., Andrews N. A., Puopolo M., Liu P. W., Jacquemont T., Pascal M. (2019). Novel
charged sodium and calcium channel inhibitor
active against neurogenic inflammation. eLife.

[63] Schmalhofer W. A., Calhoun J., Burrows R., Bailey T., Kohler M. G., Weinglass A. B., Kaczorowski G. J., Garcia M. L., Koltzenburg M., Priest B. T. (2008). ProTx-II, a Selective Inhibitor of Na_v_1.7
Sodium Channels, Blocks Action Potential Propagation in Nociceptors. Mol. Pharmacol..

[64] Delgadillo D. A., Burch J. E., Kim L. J., de Moraes L. S., Niwa K., Williams J., Tang M. J., Lavallo V. G., Khatri Chhetri B., Jones C. G. (2024). High-Throughput
Identification
of Crystalline Natural Products from Crude Extracts Enabled by Microarray
Technology and microED. ACS Central Science.

[65] Asef C. K., Vallejo D. D., Fernández F. M. (2024). Triboelectric Nanogenerators for
the Masses: A Low-Cost Do-It-Yourself Pulsed Ion Source for Sample-Limited
Applications. J. Am. Soc. Mass Spectrom..

[66] Bouza M., Li Y., Wu C., Guo H., Wang Z. L., Fernández F. M. (2020). Large-Area
Triboelectric Nanogenerator Mass Spectrometry: Expanded Coverage,
Double-Bond Pinpointing, and Supercharging. J. Am. Soc. Mass Spectrom..

[67] Bouza M., Li Y., Wang A. C., Wang Z. L., Fernández F. M. (2021). Triboelectric
Nanogenerator Ion Mobility-Mass Spectrometry for In-Depth Lipid Annotation. Anal. Chem..

[68] Jung J. H., Lee H., Kang S. S. (1996). Diacylglycerylgalactosides
from *Arisaema amurense*. Phytochemistry.

[69] Gao Z., Ali Z., Khan I. A. (2008). Glycerogalactolipids from the fruit of Lycium barbarum. Phytochemistry.

[70] Kim J. S., Shim S. H., Chae S., Han S. J., Kang S. S., Son K. H., Chang H. W., Kim H. P., Bae K. (2005). Saponins and
Other Constituents from the Leaves of *Aralia elata*. Chem. Pharm. Bull..

[71] Ma A.-C., Chen Z., Wang T., Song N., Yan Q., Fang Y.-C., Guan H.-S., Liu H.-B. (2014). Isolation of the
Molecular Species of Monogalactosyldiacylglycerols from Brown Edible
Seaweed *Sargassum horneri* and Their Inhibitory Effects
on Triglyceride Accumulation in 3T3-L1 Adipocytes. J. Agric. Food Chem..

[72] Morimoto T., Nagatsu A., Murakami N., Sakakibara J., Tokuda H., Nishino H., Iwashima A. (1995). Anti-tumour-promoting
glyceroglycolipids from the green alga, Chlorella vulgaris. Phytochemistry.

[73] Shirahashi H., Murakami N., Watanabe M., Nagatsu A., Sakakibara J., Tokuda H., Nishino H., Iwashima A. (1993). Isolation and Identification
of Anti-tumor-Promoting Principles from the Fresh-Water Cyanobacterium
Phormidium tenue. CHEMICAL & PHARMACEUTICAL
BULLETIN.

[74] Reshef V., Mizrachi E., Maretzki T., Silberstein C., Loya S., Hizi A., Carmeli S. (1997). New Acylated Sulfoglycolipids
and Digalactolipids and Related Known Glycolipids from Cyanobacteria
with a Potential To Inhibit the Reverse Transcriptase of HIV-1. J. Nat. Prod..

[75] Gustafson K. R., Cardellina J. H., Fuller R. W., Weislow O. S., Kiser R. F., Snader K. M., Patterson G. M. L., Boyd M. R. (1989). AIDS-Antiviral Sulfolipids
From Cyanobacteria (Blue-Green
Algae). JNCI: Journal of the National Cancer
Institute.

[76] Bruno A., Rossi C., Marcolongo G., Di Lena A., Venzo A., Berrie C. P., Corda D. (2005). Selective in vivo anti-inflammatory
action of the galactolipid monogalactosyldiacylglycerol. Eur. J. Pharmacol..

[77] Bergé J. P., Debiton E., Dumay J., Durand P., Barthomeuf C. (2002). In Vitro Anti-inflammatory
and Anti-proliferative Activity of Sulfolipids from the Red Alga Porphyridium
cruentum. J. Agric. Food Chem..

[78] Logvinov S.
V., Denisenko V. A., Dmitrenok P. S., Moiseenko O. P. (2012). Sulfoquinovosyldiacylglycerins
from Scaphechinus mirabilis. Chem. Nat. Compd..

[79] Wang H., Li Y.-L., Shen W.-Z., Rui W., Ma X.-J., Cen Y.-Z. (2007). Antiviral activity of a sulfoquinovosyldiacylglycerol
(SQDG) compound isolated from the green alga Caulerpa racemosa. Botanica Marina.

[80] Golik J., Dickey J. K., Todderud G., Lee D., Alford J., Huang S., Klohr S., Eustice D., Aruffo A., Agler M. L. (1997). Isolation and Structure Determination
of Sulfonoquinovosyl
Dipalmitoyl Glyceride, a P-Selectin Receptor Inhibitor from the Alga
Dictyochloris fragrans. J. Nat. Prod..

[81] Cedergren R. A., Hollingsworth R. I. (1994). Occurrence
of sulfoquinovosyl diacylglycerol in some
members of the family Rhizobiaceae. J. Lipid
Res..

[82] Sassaki G. L., Gorin P. A. J., Tischer C. A., Iacomini M. (2001). Sulfonoglycolipids
from the lichenized basidiomycete Dictyonema glabratum: isolation,
NMR, and ESI-MS approaches. Glycobiology.

[83] Chatterjee R., Singh O., Pachuau L., Malik S. P., Paul M., Bhadra K., Paul S., Kumar G. S., Mondal N. B., Banerjee S. (2010). Identification of a sulfonoquinovosyldiacylglyceride
from Azadirachta indica and studies on its cytotoxic activity and
DNA binding properties. Bioorg. Med. Chem. Lett..

[84] Ash A., Bharitkar Y. P., Murmu S., Hazra A., Ravichandiran V., Kar P. K., Mondal N. B. (2017). Ultrastructural changes in Raillietina
(Platyhelminthes: cestoda), exposed to sulfonoquinovosyldiacylglyceride
(SQDG), isolated from Neem (Azadirachta indica). Natural Product Research.

[85] Bano S., Uddin S., Ahmad V. U. (1990). An Acylated
Derivative of a New N-Acylsphingosine
from Red Alga Halymenia porphyroides. Planta
Med..

[86] Meesala S., Gurung P., Karmodiya K., Subrayan P., Watve M. G. (2018). Isolation
and structure elucidation of halymeniaol, a new antimalarial sterol
derivative from the red alga Halymenia floresii. Journal of Asian Natural Products Research.

[87] Yu H.-b., Li M., Wang W.-p., Wang X.-l. (2016). High throughput screening technologies
for ion channels. Acta Pharmacologica Sinica.

[88] Liu P., Jo S., Bean B. P. (2012). Modulation of neuronal sodium channels by the sea anemone
peptide BDS-I. Journal of Neurophysiology.

[89] Wang G. K., Strichartz G. R. (2012). State-dependent inhibition of sodium channels by local
anesthetics: A 40-year evolution. Biochemistry
(Moscow) Supplement Series A: Membrane and Cell Biology.

[90] Hurley, R. W. ; Cohen, S. P. Neuraxial Agents. In Raj’s Practical Management of Pain, Fourth ed.; Benzon, H. T. , Rathmell, J. P. , Wu, C. L. , Turk, D. C. , Argoff, C. E. , Eds.; Mosby, 2008; pp 699–713.

[91] Neubig R. R., Spedding M., Kenakin T., Christopoulos A. (2003). International
Union of Pharmacology Committee on Receptor Nomenclature and Drug
Classification. XXXVIII. Update on Terms and Symbols in Quantitative
Pharmacology. Pharmacol. Rev..

[92] Morales-Lázaro S. L., Llorente I., Sierra-Ramírez F., López-Romero A. E., Ortíz-Rentería M., Serrano-Flores B., Simon S. A., Islas L. D., Rosenbaum T. (2016). Inhibition
of TRPV1 channels by a naturally occurring omega-9 fatty acid reduces
pain and itch. Nat. Commun..

[93] Kim R.-E., Choi J.-S. (2023). Polysorbate 80 blocked
a peripheral sodium channel,
Nav1.7, and reduced neuronal excitability. Molecular
Pain.

[94] McKerrall S. J., Nguyen T., Lai K. W., Bergeron P., Deng L., DiPasquale A., Chang J. H., Chen J., Chernov-Rogan T., Hackos D. H. (2019). Structure- and Ligand-Based Discovery of Chromane
Arylsulfonamide Nav1.7 Inhibitors for the Treatment of Chronic Pain. J. Med. Chem..

[95] Wang S.-Y., Wang G. K. (2003). Voltage-gated sodium
channels as primary targets of
diverse lipid-soluble neurotoxins. Cellular
Signalling.

[96] Zhang X.-y., Bi R.-y., Zhang P., Gan Y.-h. (2018). Veratridine
modifies
the gating of human voltage-gated sodium channel Nav1.7. Acta Pharmacologica Sinica.

[97] Khatri
Chhetri B., Mojib N., Moore S. G., Delgadillo D. A., Burch J. E., Barrett N. H., Gaul D. A., Marquez L., Soapi K., Nelson H. M. (2023). Cryptic Chemical Variation
in a Marine Red Alga as Revealed by Nontargeted Metabolomics. ACS Omega.

[98] Littler, D. S. ; Littler, M. M. South Pacific Reef Plants: A Divers’ Guide to the Plant Life of South Pacific Coral Reefs; Offshore Graphics Inc., 2003.

[99] Bharti S.
K., Roy R. (2012). Quantitative
1H NMR spectroscopy. TrAC Trends
in Analytical Chemistry.

