# Supplementary material for: Discovery of Nav1.7 Inhibitors through the Screening of Marine Natural Product Extracts
Source: J Nat Prod. 2025 Oct 22;88(11):2635–44. doi: 10.1021/acs.jnatprod.5c00978 (PMC12670511; doi:10.1021/acs.jnatprod.5c00978)
Supplement: Supplementary file 1 [file np5c00978_si_001.pdf]

Supporting Information

Discovery of $Na_v1.7$ Inhibitors through the Screening of Marine Natural Product Extracts

Adetola H. Adewole[†∇], Bhuwan Khatri Chhetri[‡∇], Ghada M. Abdelwahab[†], Riya Bhanushali[†], Anne Marie Sweeney-Jones[‡], Madison Greene[†], Jaehoon Shim[⊥], Carter K. Asef[‡], Patric Vaelli[¥], Lee Barrett[⊥], Facundo M. Fernández[‡∥], Cassandra L. Quave[§], and Julia Kubanek*[†‡∥]

[∇]Both authors contributed equally

[†]School of Biological Sciences, Georgia Institute of Technology, Atlanta, Georgia 30332, United States

[‡]School of Chemistry and Biochemistry, Georgia Institute of Technology, Atlanta, Georgia 30332, United States

[⊥]F.M. Kirby Neurobiology Center, Boston Children's Hospital, and Department of Neurobiology, Harvard Medical School, Boston, Massachusetts 02115, United States

[¥]Department of Neurobiology, Harvard Medical School, Boston, Massachusetts 02115, United States

[∥]Parker H. Petit Institute for Bioengineering and Bioscience, Georgia Institute of Technology, Atlanta, Georgia 30332, United States

[§]Department of Dermatology, Center for the Study of Human Health, Emory University, Atlanta, Georgia 30322, United States

*Corresponding author's email address: julia.kubanek@biosci.gatech.edu

Figure S1:	Photographs of selected marine organisms from some of the top hits in the preliminary screen……	3
Figure S2:	MS^3 analysis of **1**……	4
Figure S3:	MS^2 analysis of **1**……	5
Figure S4:	Full MS spectrum of **1**……	6
Figure S5:	^{1}H NMR spectrum of **1** acquired at 800 MHz in DMSO-d_6……	7
Figure S6:	^{13}C NMR spectrum of **1** acquired at 200 MHz in DMSO-d_6……	8
Figure S7:	^{1}H NMR spectrum of **2** acquired at 800 MHz in DMSO-d_6……	9
Figure S8:	^{13}C NMR spectrum of **2** acquired at 200 MHz in DMSO-d_6……	10
Figure S9:	Positive ionization mode high resolution MS data for **2**……	11
Figure S10:	Voltage protocol used for human $Na_v1.7$ ($hNa_v1.7$) screening of **1** and **2**	12
Figure S11:	Primary data from Qube automated patch-clamp recordings of **1** using the Won1st voltage protocol……	13
Figure S12:	Primary data from Qube automated patch-clamp recordings of **1** using the WonPrem70 voltage protocol……	14
Figure S13:	Primary data from Qube automated patch-clamp recordings of **2** using the Won1st voltage protocol……	15
Figure S14:	Primary data from Qube automated patch-clamp recordings of **2** using the WonPrem70 voltage protocol……	16
Table S1:	Selected hits for marine natural product extract fractions active at ≤33 µg/mL in the $Na_v1.7$ thallium flux assay……	17
Table S2:	NMR spectral data for sulfoquinovosyldiacylglycerin (**2**) acquired at 800 MHz in DMSO-d_6……	18

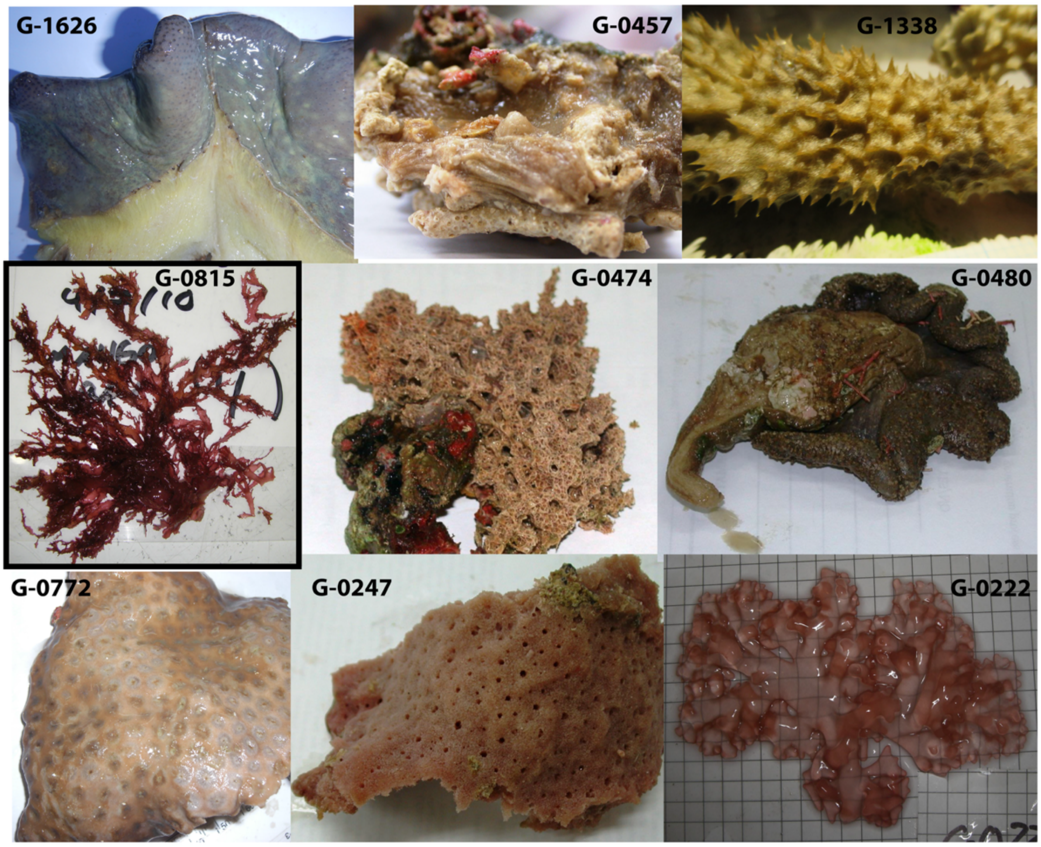

Figure S1: Photographs of selected marine organisms from some of the top hits identified in the preliminary screening, photographed at the time of collection. The organism outlined in black was selected for compound isolation. Row 1 (L–R): Soft coral *Sinularia* sp. (G-1626), unidentified member of the phylum Porifera (G-0457), another unidentified member of the phylum Porifera (G-1338); Row 2 (L–R): Red alga *Halymenia* sp. (G-0815), unidentified member of the phylum Porifera (G-0474), soft coral *Sarcophyton* sp. (G-0480); Row 3 (L–R): Zoanthid *Palythoa* sp. (G-0772), sponge *Cribrochalina* sp. (G-0247), red alga *Gibsmithia hawaiiensis* (G-0222).

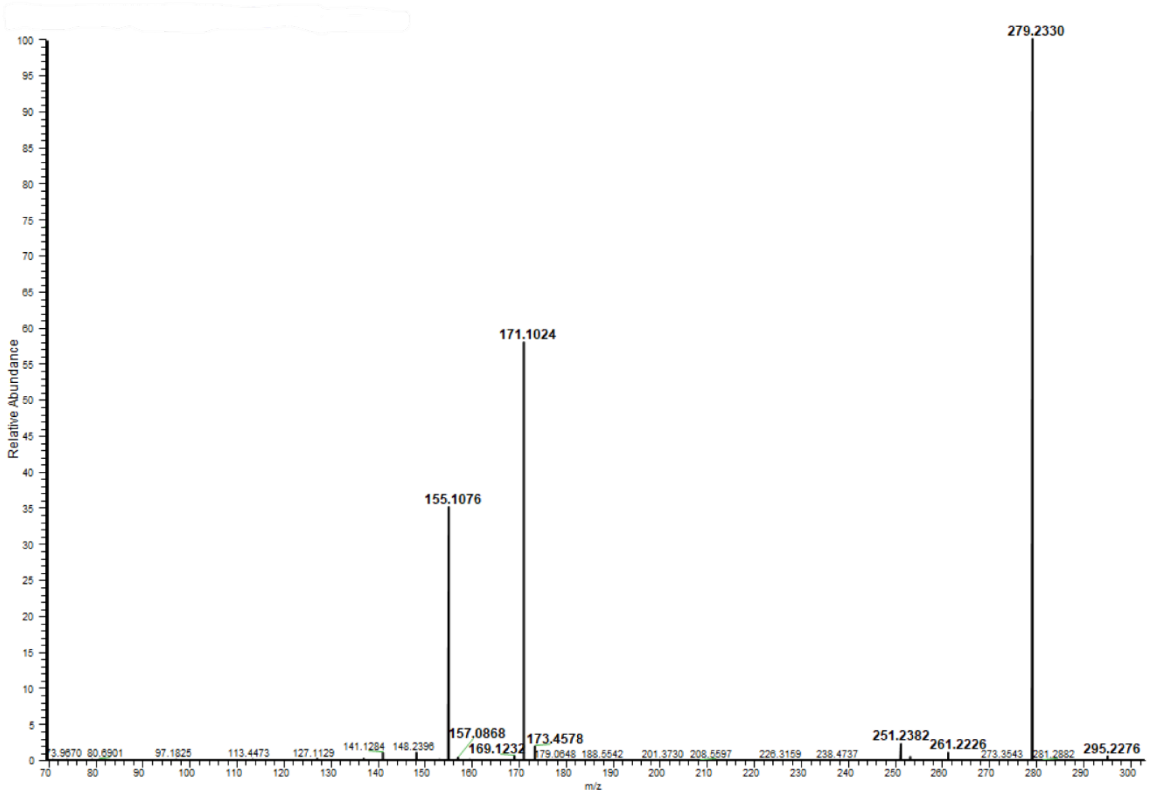

Figure S2: MS^3 analysis of **1.** The singly oxidized precursor ion at *m/z* 831.6, generated using triboelectric nanogenerator (TENG) nanoelectrospray ionization (nanoESI) in the negative ionization mode, was fragmented using a collision-induced dissociation (CID) energy of 45V. The oxidized species of the C18:1 fragment at *m/z* 297.2 was subsequently selected and further fragmented with a CID energy of 35V. Resulting fragments, scanned from *m/z* 70 to 300, revealed diagnostic ions consistent with the presence of double bonds at the Δ9 position on the 18:1 chain.

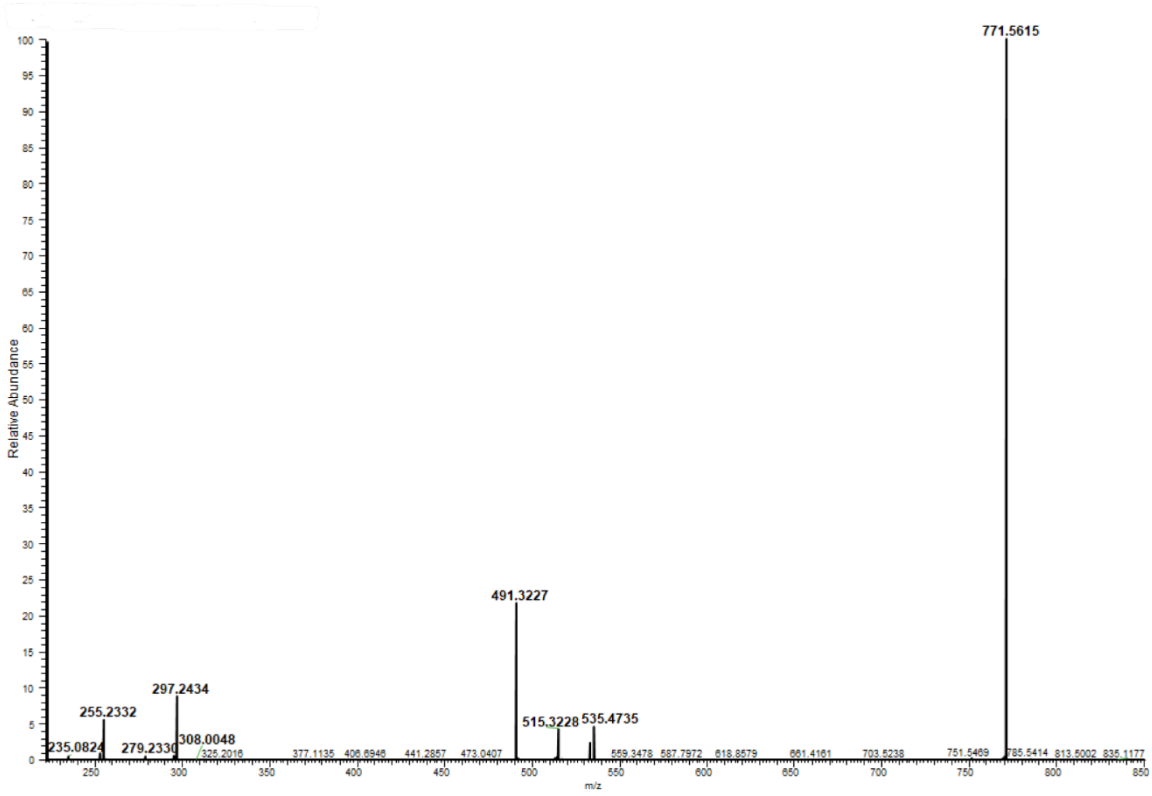

Figure S3: MS^2 analysis of **1**. The fragmentation of the singly oxidized precursor ion at *m/z* 831.6 in negative mode via TENG nanoESI with CID energy of 45V, displaying product ions scanned between *m/z* 224 and 842.

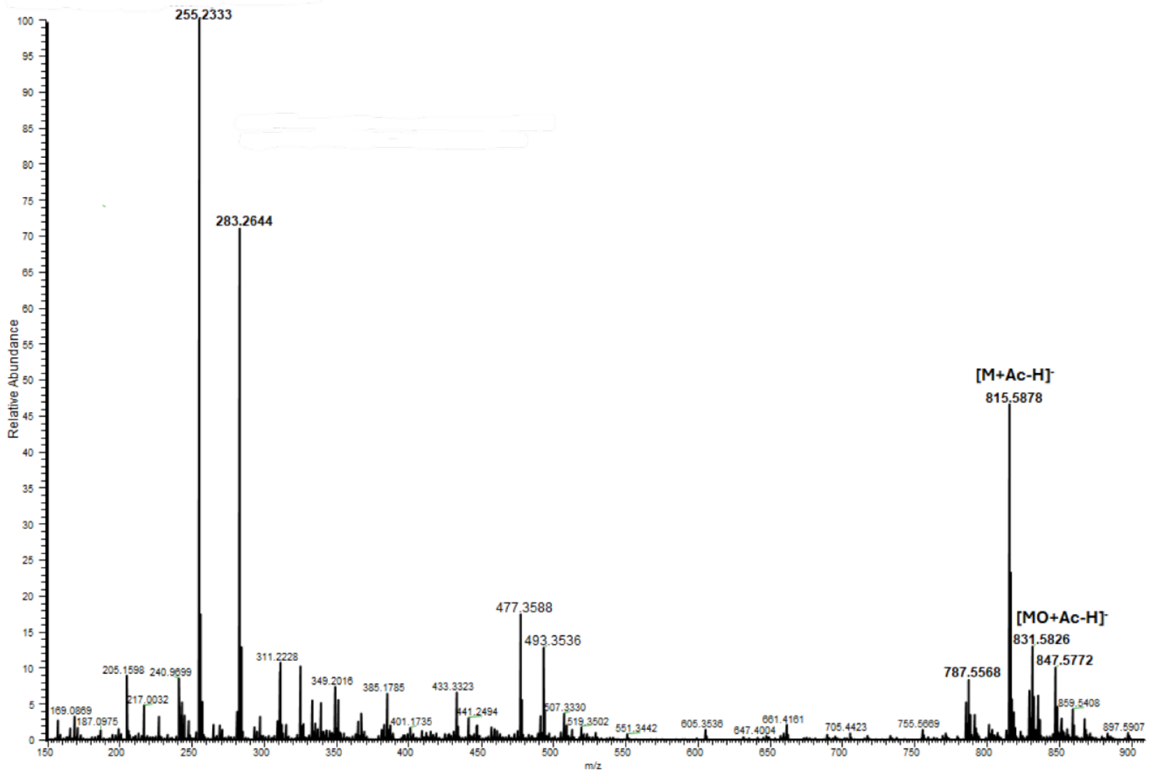

Figure S4: Full MS spectrum of **1** (250 µM) analyzed using TENG nanoESI in the negative ionization mode. The detected species are labeled, with MO representing the singly oxidized glycoglycerolipid formed through epoxidation and Ac denoting acetate. Background contaminants, including palmitic acid (*m/z* 255.2333) and stearic acid (*m/z* 283.2644), commonly originate from glassware and solvents.

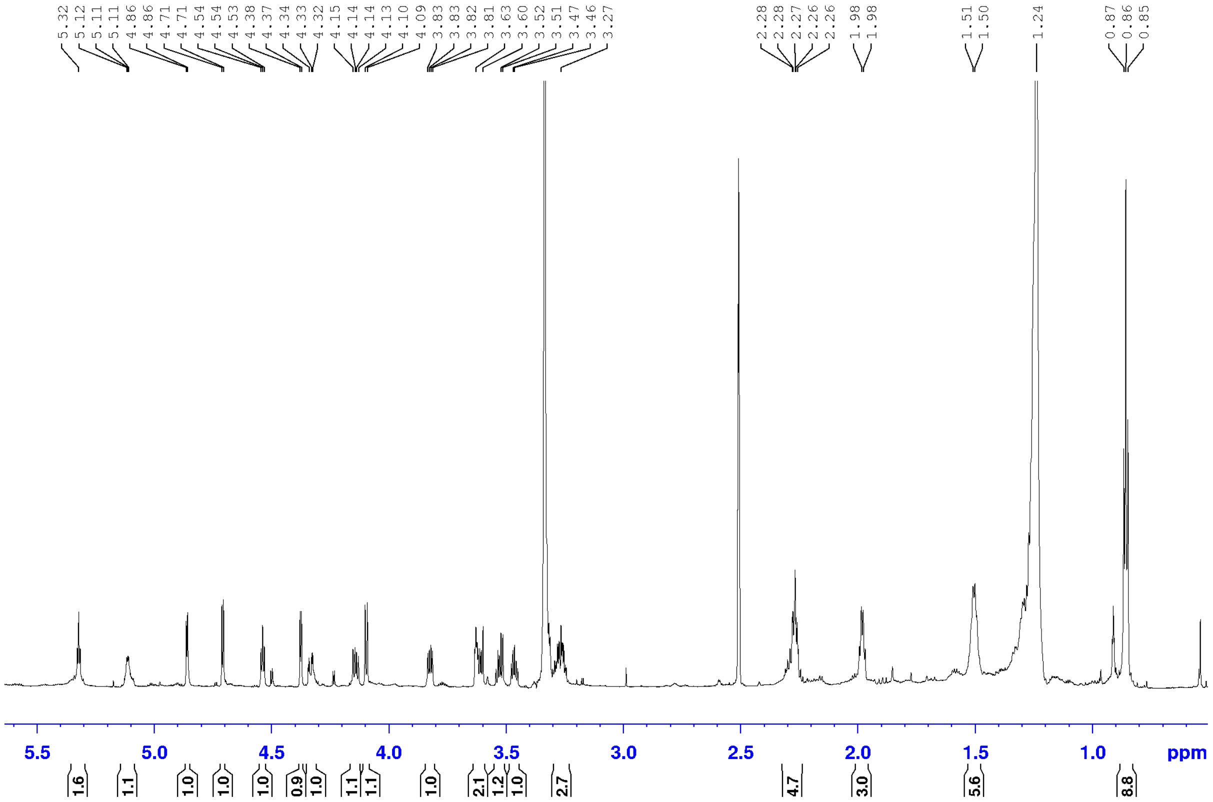

Figure S5: ^{1}H NMR spectrum of **1** acquired at 800 MHz in DMSO-d_6. The specific rotation of **1** was determined as $[\alpha]^{25}_{D}$ +2.5 (c 0.068, MeOH).

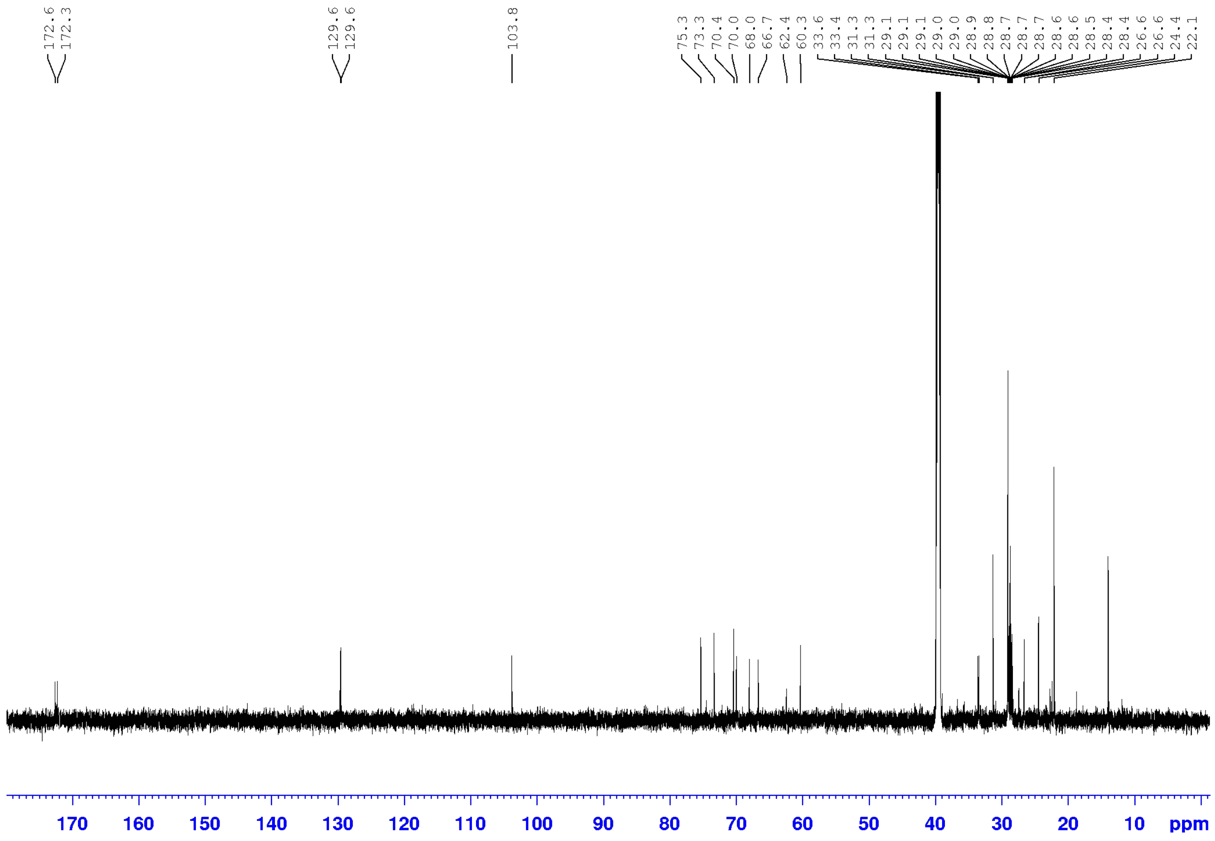

Figure S6: ^{13}C NMR spectrum of **1** acquired at 200 MHz in DMSO-d_6.

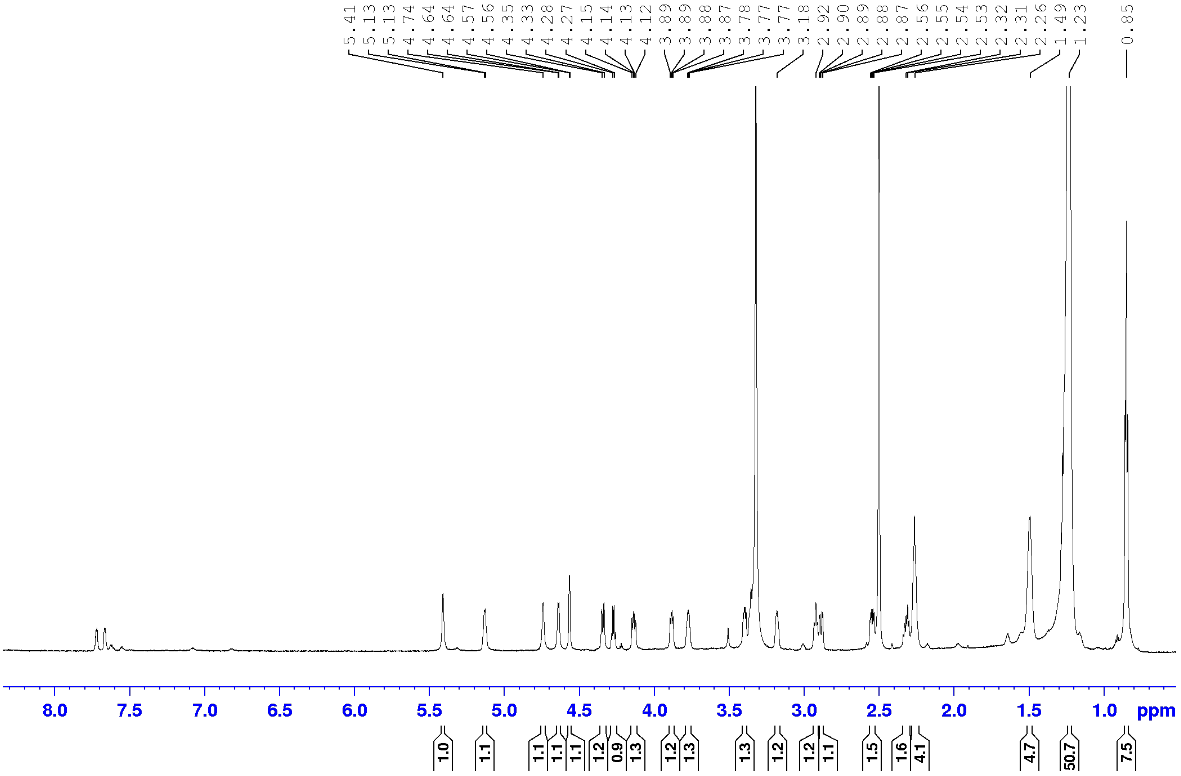

Figure S7: ^{1}H NMR spectrum of **2** acquired at 800 MHz in DMSO-d_6. The signals at δ_H 7.66 and 7.71 ppm belong to phthalate impurities.

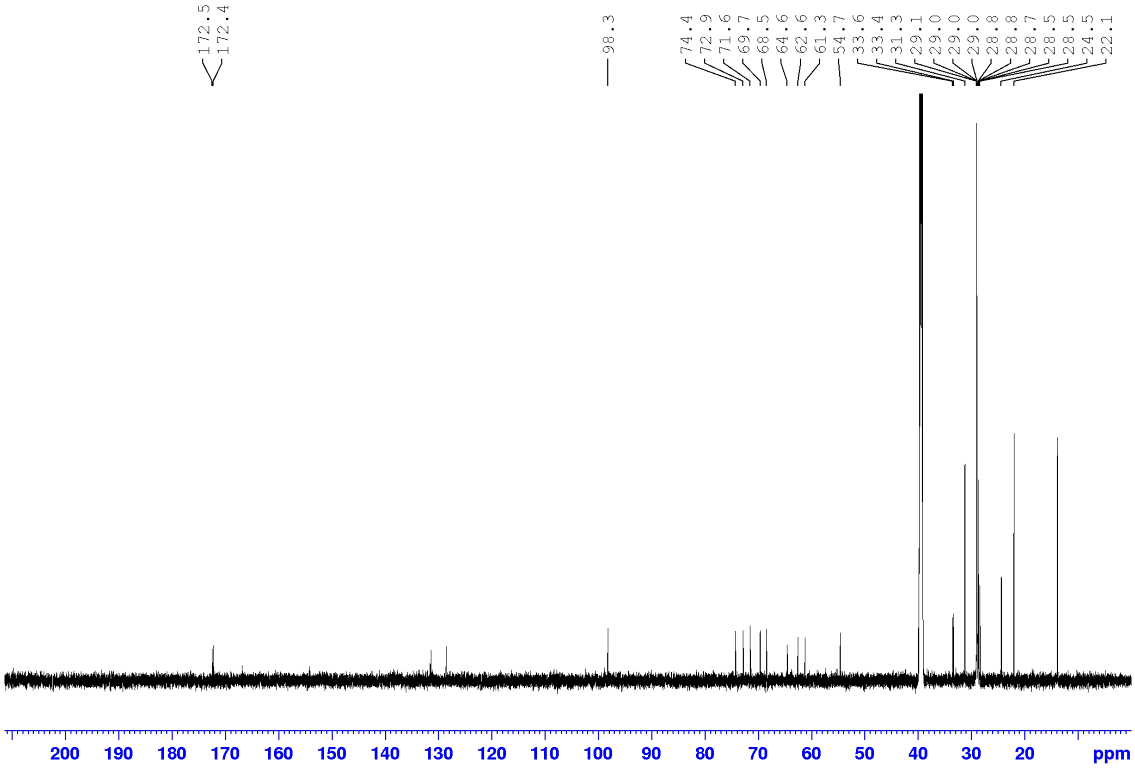

Figure S8: ^{13}C NMR spectrum of **2** acquired at 200 MHz in DMSO-d_6. The signals at δ_C 128.6 and 131.5 ppm belong to phthalate impurities.

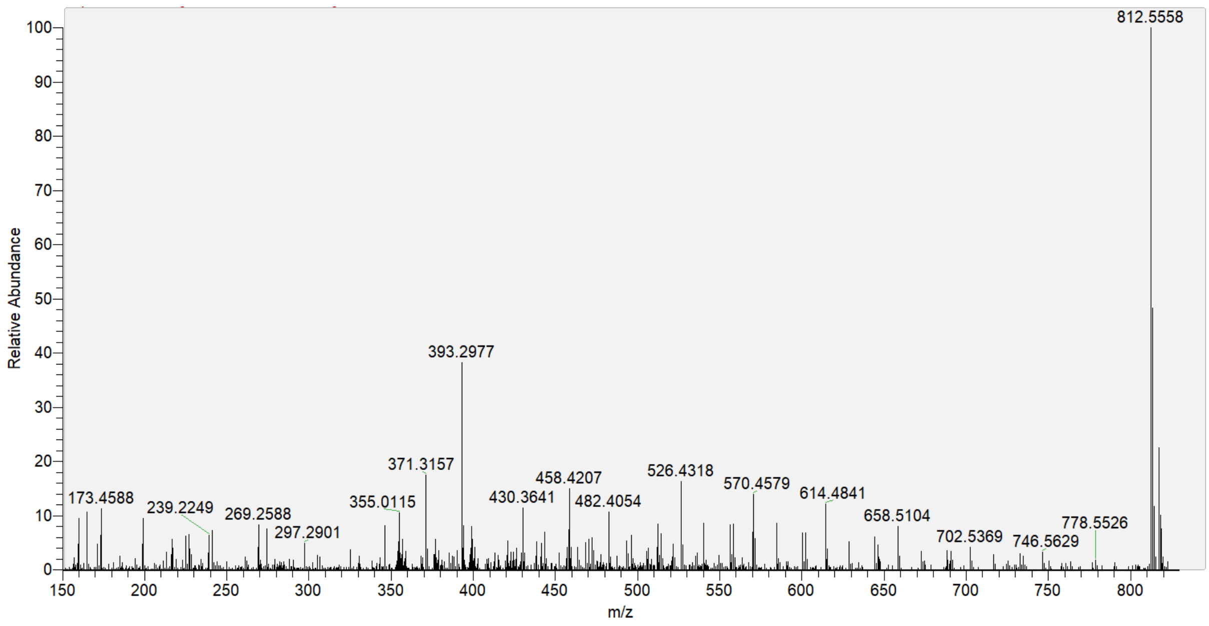

Figure S9: Positive ionization mode HRMS data for **2**

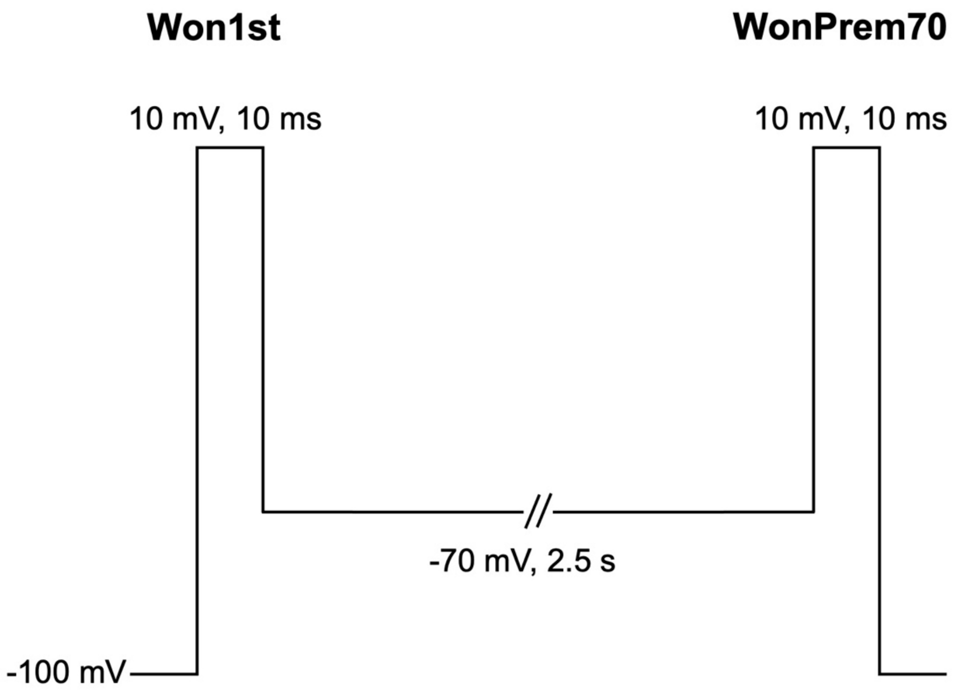

Figure S10: Voltage protocol steps used for human $Na_v1.7$ ($hNa_v1.7$) screening of **1** and **2**. Won1st represents the first voltage step (-100 mV holding potential) for measuring tonic block, and WonPrem70 corresponds to the state-dependent block step with a holding potential of -70 mV prior to the test pulse.

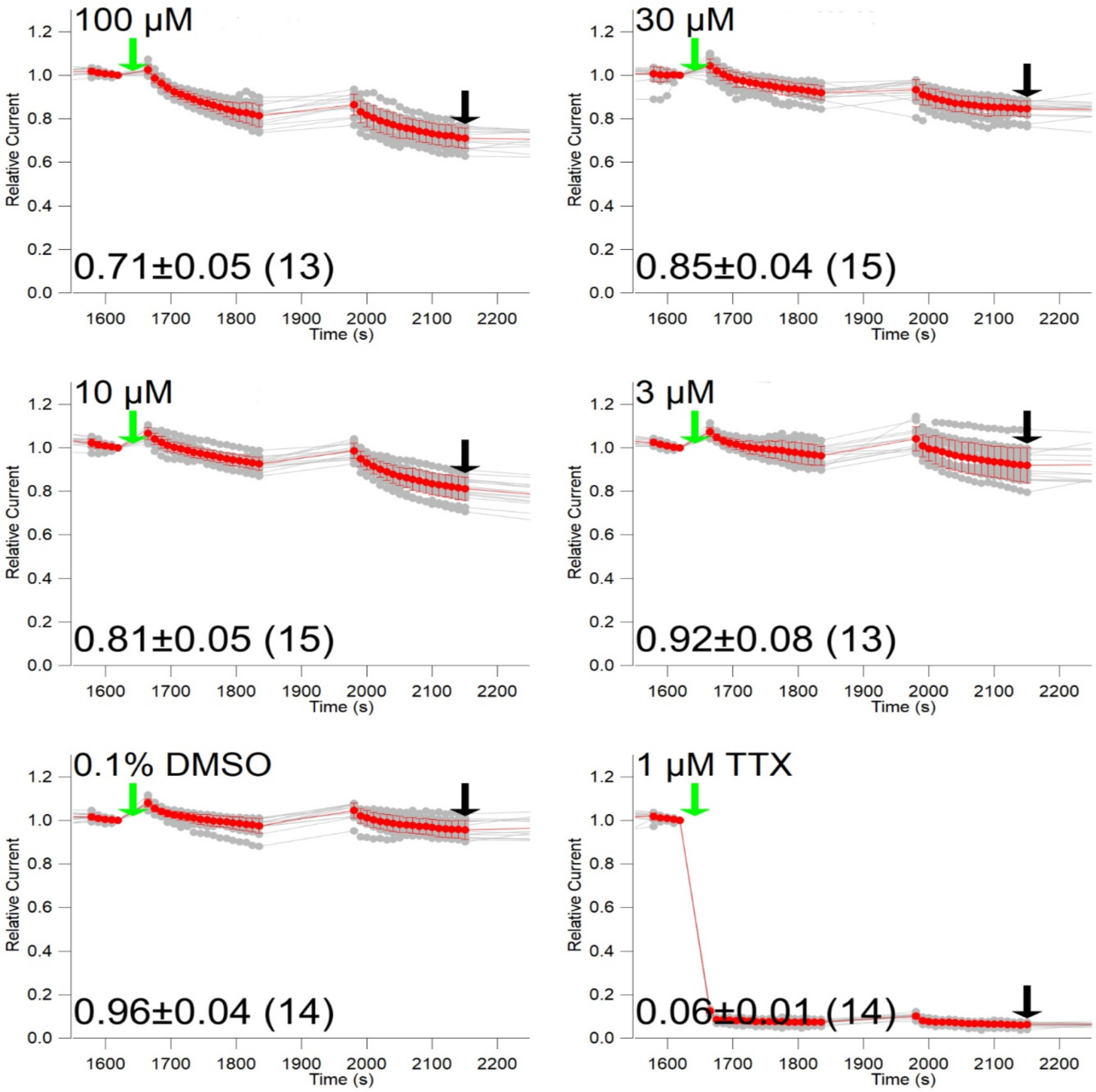

Figure S11: Primary data from Qube automated patch-clamp recordings of **1** using the Won1st voltage protocol. The plots show normalized Na^+ channel currents before (green arrow) and after (black arrow) compound application. Data points between the arrows represent the time course of inhibition, where a greater current decline indicates stronger ion channel inhibition. The number in the lower-left corner of each plot represents the inhibition ratio (black arrow/green arrow) ± standard deviation and sample size in brackets. Inhibition is more potent at -70 mV than at -100 mV, reflecting increased potency in the inactivated state.

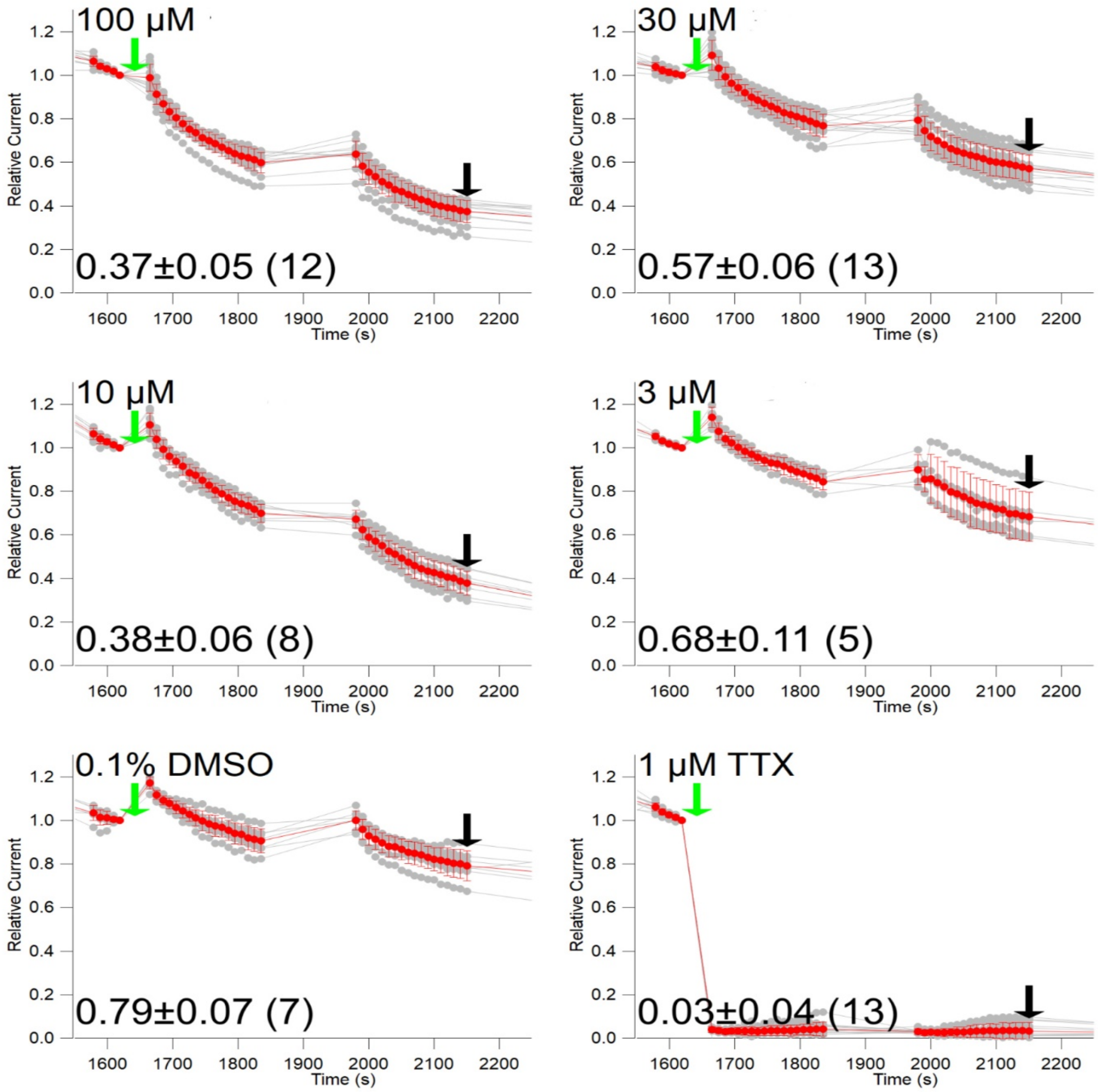

Figure S12: Primary data from Qube automated patch-clamp recordings of **1** using the WonPrem70 voltage protocol. The plots show normalized Na^+ channel currents before (green arrow) and after (black arrow) compound application. Data points between the arrows represent the time course of inhibition, where a greater current decline indicates stronger ion channel inhibition. The number in the lower-left corner of each plot represents the inhibition ratio (black arrow/green arrow) ± standard deviation and sample size in brackets. Inhibition is more potent at -70 mV than at -100 mV, reflecting increased potency in the inactivated state.

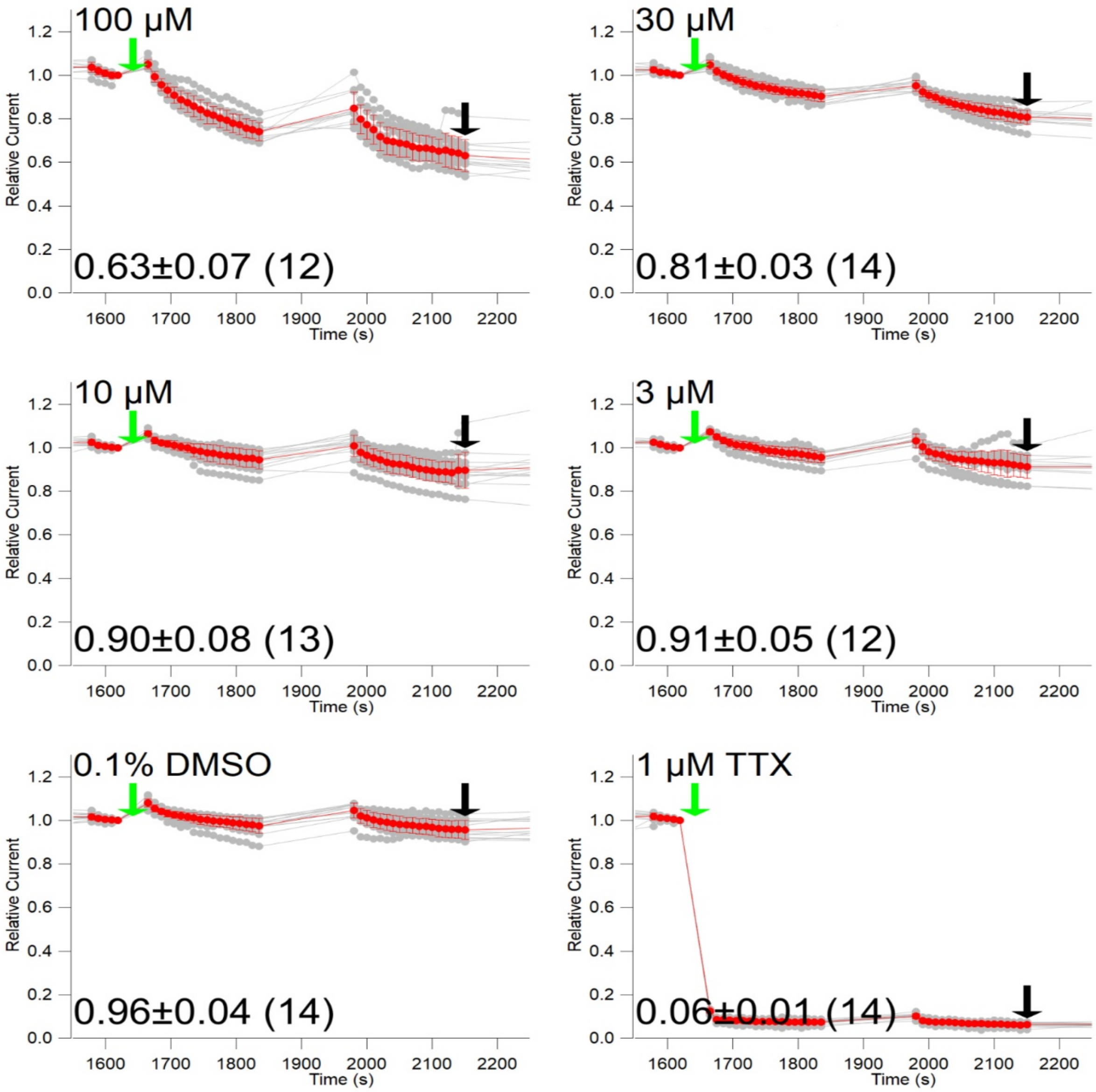

Figure S13: Primary data from Qube automated patch-clamp recordings of **2** using the Won1st voltage protocol. The plots show normalized Na^+ channel currents before (green arrow) and after (black arrow) compound application. Data points between the arrows represent the time course of inhibition, where a greater current decline indicates stronger ion channel inhibition. The number in the lower-left corner of each plot represents the inhibition ratio (black arrow/green arrow) ± standard deviation and sample size in brackets. Inhibition is more potent at -70 mV than at -100 mV, reflecting increased potency in the inactivated state.

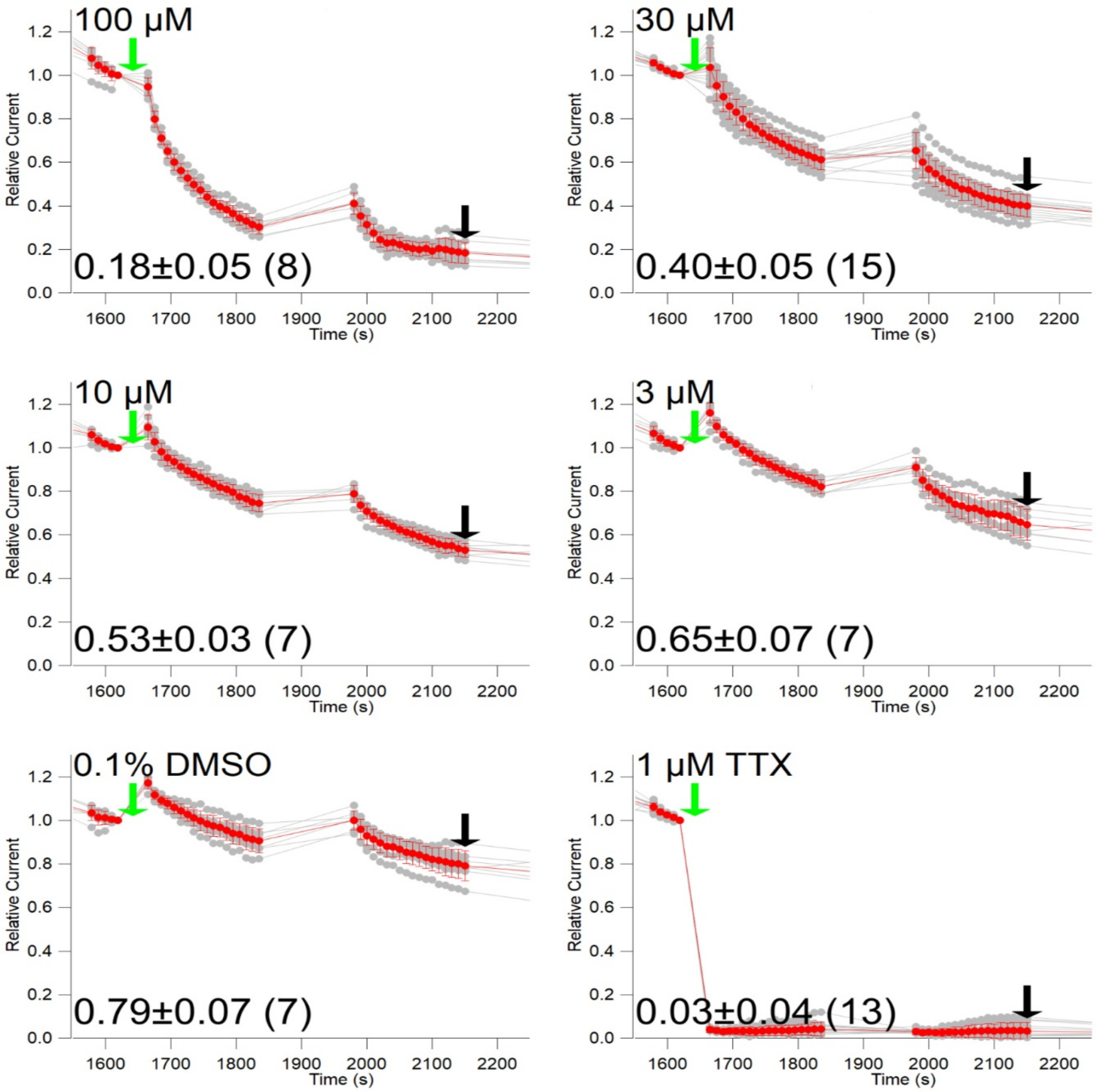

Figure S14: Primary data from Qube automated patch-clamp recordings of **2** using the WonPrem70 voltage protocol. The plots show normalized Na^+ channel currents before (green arrow) and after (black arrow) compound application. Data points between the arrows represent the time course of inhibition, where a greater current decline indicates stronger ion channel inhibition. The number in the lower-left corner of each plot represents the inhibition ratio (black arrow/green arrow) ± standard deviation and sample size in brackets. Inhibition is more potent at -70 mV than at -100 mV, reflecting increased potency in the inactivated state.

Table S1: Selected hits for marine natural product extract fractions active at ≤33 µg/mL in the $Na_v1.7$ thallium flux assay. EtOAc = ethyl acetate-soluble fraction; DCM = dichloromethane-soluble fraction.

	**Code**	**Organism**	**Fraction**	**Collection site**	**Latitude**	**Longitude**
1	G-0222	*Gibsmithia hawaiiensis*	EtOAc	Sovu, Northern Lau, Fiji	-17.1548	-178.812
2	G-0247	*Cribrochalina* sp.	EtOAc	Kibobo, Northern Lau, Fiji	-17.0514	-179.055
3	G-0457	Unidentified member in the phylum Porifera	EtOAc	Wakaya, Fiji	-17.6148	178.974
4	G-0474	Unidentified member in the phylum Porifera	EtOAc	Makogai, Fiji	-17.4648	178.906
5	G-0480	*Sarcophyton* sp.	EtOAc	Makogai, Fiji	-17.442	178.952
6	G-0772A	*Palythoa* sp.	EtOAc	Suva, Viti Levu, Fiji	-18.1858	178.512
7	G-0815	*Halymenia* sp.	DCM	Mango Bay, Viti Levu, Fiji	-18.2364	177.781
8	G-0815	*Halymenia* sp.	EtOAc	Mango Bay, Viti Levu, Fiji	-18.2364	177.781
9	G-1338	Unidentified member in the phylum Porifera	EtOAc	Western province, Solomon Islands	*	*
10	G-1626	*Sinularia* sp.	Aqueous	Nalova bay, Nacula, Fiji	-16.9126	177.379
11	G-1626	*Sinularia* sp.	EtOAc	Nalova bay, Nacula, Fiji	-16.9126	177.379

*Specific coordinates were not recorded

Table S2: NMR spectral data for sulfoquinovosyldiacylglycerin (**2**) acquired at 800 MHz in DMSO- d_6.

Position #	δ_C	$\delta_{H\ (mult,\ J\ Hz)}$	COSY	HMBC
1a	62.6 (CH_2)	4.13 dd (11.8, 7.4)	H-2	C-2, C-3, C-16′′
1b		4.35 dd (11.8, 2.7)	H-2	C-3
2	69.7 (CH)	5.13 m	H-1, H-3a, H-3b	C-3, C-16′
3a	64.6 (CH_2)	3.39 dd (10.6, 5.7)	H-2	C-1, C-2, C-1′′′
3b		3.88 dd (10.6, 5.9)	H-2	C-1, C-2, C-1′′′
1′	13.9 (CH_3)	0.85 t (6.7)	H-2′	C-2′, C-3′
1′′	13.9 (CH_3)	0.85 t (6.7)	H-2′′	C-2′′, C-3′′
2a′	22.1 (CH_2)	1.28 m	H-1′	C-1′
2b′		1.28 m	H-1′	C-1′
2a′′	22.1 (CH_2)	1.28 m	H-1′′	C-1′
2b′′		1.28 m	H-1′′	C-1′
3a′	31.3 (CH_2)	1.23 m		
3b′		1.23 m		
3a′′	31.3 (CH_2)	1.23 m		
3b′′		1.23 m		
4a′–13a′	28.5–29.1 (CH_2)	1.23 br s	14a′, 14b′	C-14′
4b′–13b′		1.23 br s	14a′, 14b′	C-14′
4a′′–13a′′	28.5–29.1 (CH_2)	1.23 br s	14a′′, 14b′′	C-14′′
4b′′–13b′′		1.23 br s	14a′′, 14b′′	C-14′′
14a′	24.5 (CH_2)	1.49 m	H-13a′, H-13b′	C-13′, C-15′, C-16′
14b′		1.49 m	H-13a′, H-13b′	C-13′, C-15′, C-16′
14a′′	24.5 (CH_2)	1.49 m	H-13a′′, H-13b′′	C-13′′, C-15′′, C-16′′
14b′′		1.49 m	H-13a′′, H-13b′′	C-13′′, C-15′′, C-16′′
15a′	33.4/33.6 (CH_2)	2.24–2.34 m	H-14a′, H-14b′	C-13′, C-14′, C-16′
15b′		2.24–2.34 m	H-14a′, H-14b′	C-13′, C-14′, C-16′
15a′′	33.4/33.6 (CH_2)	2.24–2.34 m	H-14a′′, H-14b′′	C-13′′, C-14′′, C-16′′
15b′′		2.24–2.34 m	H-14a′′, H-14b′′	C-13′′, C-14′′, C-16′′
16′	172.4 (C)			
16′′	172.5 (C)			
1′′′	98.3 (CH)	4.56 d (3.7)	H-2′′′	C-3
2′′′	71.6 (CH)	3.18 dd (9.5, 3.7)	H-1′′′, H-3′′′, OH (4.64)	C-3′′′
3′′′	72.9 (CH)	3.35 (overlaid)	H-2′′′, H-4′′′, OH (4.74)	C-2′′′, C-4′′′
4′′′	74.3 (CH)	2.92 dd (9.0, 9.0)	H-3′′′, H-5′′′, OH (5.41)	C-3′′′, C-5′′′, C-6′′′
5′′′	68.5 (CH)	3.78 m	H-4′′′, H-6a′′′, H-6b′′′	C-4′′′, C-6′′′
6a′′′	54.7 (CH_2)	2.55 dd (13.9, 6.4)	H-5′′′	C-4′′′, C-5′′′
6b′′′		2.89 dd (13.9, 4.7)	H-5′′′	C-4′′′, C-5′′′
	OH	4.64 d (5.5)	H-2′′′	C-2′′′, C-3′′′
	OH	4.74 br s	H-3′′′	C-2′′′, C-3′′′
	OH	5.41 br s	H-4′′′	C-3′′′, C-5′′′
